# Supplementary material for: Use of routine death and illness surveillance data to provide insight for UK pandemic planning: lessons from COVID-19
Source: BMJ Open. 2021 Feb 8;11(2):e044707. doi: 10.1136/bmjopen-2020-044707 (PMC7871230; doi:10.1136/bmjopen-2020-044707)
Supplement: Author's manuscript [file bmjopen-2020-044707.draft_revisions.pdf]

BMJ Open is committed to open peer review. As part of this commitment we make the peer review history of every article we publish publicly available.

When an article is published we post the peer reviewers' comments and the authors' responses online. We also post the versions of the paper that were used during peer review. These are the versions that the peer review comments apply to.

The versions of the paper that follow are the versions that were submitted during the peer review process. They are not the versions of record or the final published versions. They should not be cited or distributed as the published version of this manuscript.

BMJ Open is an open access journal and the full, final, typeset and author-corrected version of record of the manuscript is available on our site with no access controls, subscription charges or pay-per-view fees (http://bmjopen.bmj.com).

If you have any questions on BMJ Open's open peer review process please email info.bmjopen@bmj.com

BMJ Open

Understanding the context of illness and death – critical information for COVID-19 response planning

Journal:	*BMJ Open*
Manuscript ID	bmjopen-2020-044707
Article Type:	Original research
Date Submitted by the Author:	14-Sep-2020
Complete List of Authors:	Clough, Helen; University of Liverpool Faculty of Health and Life Sciences, Institute of Infection, Veterinary and Ecological Sciences<br>McIntyre, Kirsty; University of Liverpool Faculty of Health and Life Sciences, Institute of Infection, Veterinary and Ecological Sciences<br>Patterson, Grace; University of Liverpool Faculty of Health and Life Sciences, Institute of Infection, Veterinary and Ecological Sciences<br>Harris, John; University of Liverpool, Institute of Population Health Sciences<br>Rushton, Jonathan; University of Liverpool Faculty of Health and Life Sciences, Institute of Infection, Veterinary and Ecological Sciences
Keywords:	STATISTICS & RESEARCH METHODS, EPIDEMIOLOGY, COVID-19, PUBLIC HEALTH

SCHOLARONE™
Manuscripts

I, the Submitting Author has the right to grant and does grant on behalf of all authors of the Work (as defined in the below author licence), an exclusive licence and/or a non-exclusive licence for contributions from authors who are: i) UK Crown employees; ii) where BMJ has agreed a CC-BY licence shall apply, and/or iii) in accordance with the terms applicable for US Federal Government officers or employees acting as part of their official duties; on a worldwide, perpetual, irrevocable, royalty-free basis to BMJ Publishing Group Ltd ("BMJ") its licensees and where the relevant Journal is co-owned by BMJ to the co-owners of the Journal, to publish the Work in this journal and any other BMJ products and to exploit all rights, as set out in our licence.

The Submitting Author accepts and understands that any supply made under these terms is made by BMJ to the Submitting Author unless you are acting as an employee on behalf of your employer or a postgraduate student of an affiliated institution which is paying any applicable article publishing charge ("APC") for Open Access articles. Where the Submitting Author wishes to make the Work available on an Open Access basis (and intends to pay the relevant APC), the terms of reuse of such Open Access shall be governed by a Creative Commons licence – details of these licences and which Creative Commons licence will apply to this Work are set out in our licence referred to above.

Other than as permitted in any relevant BMJ Author's Self Archiving Policies, I confirm this Work has not been accepted for publication elsewhere, is not being considered for publication elsewhere and does not duplicate material already published. I confirm all authors consent to publication of this Work and authorise the granting of this licence.

For peer review only

Title

Understanding the context of illness and death – critical information for COVID-19 response planning

Authors

Corresponding author

Name: Helen E Clough

Address: Department of Livestock and One Health, Institute of Infection, Veterinary and Ecological Sciences, University of Liverpool, Leahurst Campus, Neston, Cheshire, CH64 7TE, UK.

Email: h.e.clough@liverpool.ac.uk

Other authors

Name: K Marie McIntyre

Address: Department of Livestock and One Health, Institute of Infection, Veterinary and Ecological Sciences, University of Liverpool, Leahurst Campus, Neston, Cheshire, CH64 7TE, UK.

Name: Grace E Patterson

Address: Department of Livestock and One Health, Institute of Infection, Veterinary and Ecological Sciences, University of Liverpool, Leahurst Campus, Neston, Cheshire, CH64 7TE, UK.

Name: John P Harris

Address: Department of Public Health and Policy, Institute of Population Health Sciences, University of Liverpool, Liverpool, United Kingdom L69 3BX, UK.

Name: Jonathan Rushton

Address: Department of Livestock and One Health, Institute of Infection, Veterinary and Ecological Sciences, University of Liverpool, IC2 Building, 146 Brownlow Hill, Liverpool, L3 5RF, UK.

Word count excluding title page, abstract, references, figures and tables: 3,027 words.

For peer review only

ABSTRACT

Objectives

Reporting of COVID-19 cases, deaths, and testing has often lacked context for appropriate assessment of disease burden within risk groups. The research considers how routine surveillance data might be used to provide early insight into a pandemic and identify risk factors, setting COVID-19 deaths early on in the pandemic in context. This will facilitate the anticipation of wider consequences of a pandemic from the earliest stage, reducing fear, aiding in accurately assessing disease burden and in ensuring appropriate disease mitigation.

Setting

United Kingdom, 2020.

Participants

The study is a secondary analysis of routine, public-domain, surveillance data and information from ONS, NHS111 and PHE on deaths and disease.

Primary and secondary outcome measures

Our principal focus is ONS data on deaths which mention COVID-19 on the death certificate. We also consider information provided in NHS111 and PHE data summaries.

Results

Deaths with COVID-19 significantly (but not entirely) contributed to abnormally elevated all-cause mortality in the UK from weeks 12-18 of 2020. It was apparent early that COVID-19 is the greatest threat to those with underlying illness, rarely endangering people aged under 40 years. COVID-19 related death rates differ by region, possibly reflecting underlying population structure. Risk of COVID-19-related death is greater for health and social care staff and BAME individuals, having allowed for documented risk factors.

Conclusion

Early contextualization of public health data is critical to understand who gets sick, when, and why. Understanding at-risk groups facilitates a targeted response considering indirect consequences of society's reaction to a pandemic alongside disease-related impacts. COVID-19 related deaths mainly mirror historical patterns, and excess non-COVID-19 related deaths partly

For peer review only

reflect reduced access to and uptake of healthcare during lockdown. Future outbreak response will improve through better understanding of connectivity between disease monitoring systems to aid interpretation of disease risk patterns, facilitating nuanced mitigation measures.

Article summary

Strengths and limitations

- The study shows how routine, public domain data can be used to provide pertinent insight into a pandemic in even its earliest stages
- The use of imaginative approaches to graphical display and numerical commentary ensures that the work can be understood by readers without a statistical specialism
- This study uses a freely available statistics package to explore public domain data sets, ensuring that results are both transparent and repeatable
- Insight is limited by problems in identifying raw data from some sources: improving ease of access will strengthen this process and improve the relevance of future inferences.

Keywords

Statistics and research methods; epidemiology; COVID-19; public health

For peer review only

INTRODUCTION

Intense media reporting during the COVID-19 pandemic has focused on presenting daily data on cases, deaths and testing associated with the virus. The pandemic has undoubtedly changed our world - governments have employed unprecedented (in our times) lockdown methods to reduce transmission. These measures have greatly impacted society. The regular reporting of daily COVID-19 infections and deaths has alarmed the public, particularly when understanding of risk factors dictating severity of COVID-19 symptoms is only slowly emerging. The global population is immunologically naïve to this emerging pathogen, and society has no available specific mitigation measures including immunological therapies, other than hand washing, social distancing, mask wearing etc. Clinical and support staff in hospitals, health and social care staff in care homes and other settings, and key workers in transport and infrastructure industries are at increased risk of contracting the disease[1] due to frequent contact with people with high viral loads and the high aerosolized and fomite transmission potential of this virus[2]. Early analyses in England and Wales identified main risk factors for death from COVID-19 including older age, deprivation, and comorbidities[3], but did not consider how this risk differed from 'typical' all-cause mortality among these groups. This baseline comparison is vital to understanding what additional risk is posed by COVID-19, and to whom. Excess mortality from COVID-19 in the UK has been modelled controlling for underlying conditions and age[4], and some conditions such as uncontrolled diabetes and severe asthma are associated with death[3]. However, understanding of the health loss impacts of COVID-19 is still limited by a lack of contextualising information, reducing our ability to respond to the challenges this disease poses, both directly and indirectly, in a proportionate, targeted manner.

We provide context for deaths and disease from COVID-19, by comparing these against a historical benchmark of when, who, and how people become ill and died pre-COVID-19. Examining associations between poor COVID-19 outcomes, demographic and socio-economic differences, age, sex and comorbidities in the context of 'usual' population health structures, enhances understanding of specific risk groups and hence has a role to play in maximising the effectiveness of risk mitigation strategies whilst minimizing the likelihood of unnecessary and undesirable impacts. Examining excess deaths (above that normally expected at a time-point) is futhermore important for interpreting the total impact of a pandemic. Syndromic surveillance describing clinical symptoms and healthcare use is scrutinised to supplement clinical surveillance information used to populate the COVID-19 epidemic curve.

For peer review only

METHODS

Data sources

The principal data source was the Office for National Statistics (ONS); dashboards from Public Health England (PHE) and syndromic surveillance in England via NHS 111 were additionally consulted. Primary focus for the analyses presented was the ONS data, which provide gold-standard confirmed recorded causes of death for UK residents. Use of ONS data is licensed under the Open Government Licence v.3.0.

Statistical methods

Profiles for COVID-19 were considered against systematic differences in historical disease rates, to identify when disease was in excess of expected rates. Where possible, current disease rates were compared against historic baselines and/or adjusted for differing demographic factors such as socio-economic deprivation and underlying population density. Temporal and geographic comparisons were made between different risk factor groups.

RESULTS

Temporal variation in COVID-19 deaths

The number of deaths from all causes varies annually and seasonally, peaking in Winter with an average of 9,863 (standard deviation (SD)=1,269) deaths occurring weekly in England and Wales between January 2010 and December 2019. Typically, respiratory deaths range from 10% to 22% of all deaths and are seasonal, peaking annually in January; the 2015 peak was atypically high (16,237 deaths in week two compared to an average of 12,277 deaths that week over the previous five years) due to a severe influenza season. Minimum weekly deaths over this five years are 6,606 (week 54, 2013). In 2020 deaths from respiratory infections were as expected until early April (week 14), after which they became higher than historical rates when including deaths from COVID-19 (Figure 1a) (in week 14 observed respiratory deaths exceeded the historical upper 95% confidence interval limit by 260). An excess of unexplained deaths becomes clear from week 14 onwards (Figure 1b).

For peer review only

Regional differences in COVID-19 deaths

Regions of England and Wales experience different death rates and this pattern is true for deaths from COVID-19 (Figure 2) e.g. rates were highest and peaked in week 17 in London (204 per million), the North-West (185 per million), the North-East (179 per million), and the West Midlands (169 per million). Peak rates were lowest in the South-West (95 per million) and East Midlands (116 per million). From weeks 13-18 (March 23-May 3), all regions of England and Wales experienced excess non-COVID-19 related deaths. This was most apparent in the West Midlands in week 17 (starting April 20), with a peak of approximately 91 deaths per million. Between weeks 13-18 (March 23- May 3) there were 46,594 excess deaths in England and Wales, 13,399 of which were listed as non-COVID-19-related. In week 25 (19th June) the total deaths dips below the 5-year historical average for the first time (9,339 compared to 9,404) and this pattern continued until 10th July (most recent data at time of writing).

Occupational differences in COVID-19 deaths

After age-standardisation, men employed in lower skilled occupations (21.4, 95% CI 18.6-24.2) (Figure 3a) were more likely to die of COVID-related illness, as was true for all-cause mortality (Figure 3b). This differs for women, where those employed as carers in health and social care, leisure and other service operations (Figure 3a) were most likely to die from COVID-related illness, but not more likely to die if examining all-cause mortality (Figure 3b). For both men and women, the less technical and more manual occupation (using ONS Standard Occupation Classification (SOC) 2020 categories), the greater the risk of dying from any cause including COVID-19-related disease. In addition, as occupations become more manual, variation in age-standardised mortality rates is higher, particularly for men or women in certain SOC categories e.g. men undertaking administrative and secretarial roles; women in skilled trades; men in caring, leisure and other service occupations; men in sales and customer service roles; women working as process, plant and machine operatives; and men undertaking low skilled elementary roles. A crude comparison suggests that age-standardised mortality rates for most occupations are reduced by COVID-19 relative to deaths from any cause (Table 1), with the rate only increased (for both sexes) in caring, leisure and other service occupations.

For peer review only

SOC	Group	Females, COVID	Females, All	Males, COVID	Males, All
1	Managers, directors and senior officials (baseline)				
2	Professional occupations	1.05	1.02	0.67	0.72
3	Associate professional & technical occupations	0.85	0.87	0.89	1.07
4	Administrative & secretarial occupations	0.95	1.14	1.65	1.61
5	Skilled trades occupations	1.60	2.06	1.39	2.18
6	Caring, leisure & other service occupations	1.88	1.77	2.13	1.98
7	Sales & customer service occupations	1.35	1.37	1.70	1.49
8	Process, plant & machine operatives	1.43	2.36	1.85	1.97
9	Low skilled elementary occupations	1.53	1.66	2.55	2.77

Table 1: Ratio of estimated age-standardised mortality rates comparing occupational categories with a baseline of managerial workers (SOC group 1) for (a) COVID-19 associated male and female deaths; and (b) all-cause male and female deaths (including COVID-19)[i]

Ethnic associations with COVID-19 deaths

As previously reported by the ONS[5] there are increased odds of dying from COVID-19 for Bangladeshi/Pakistani, Black and Indian ethnic groups (for both sexes) when compared with a baseline white group and adjusted for age, region, rural/urban, Index of Multiple Deprivation decile, household composition, socio-economic status and underlying health. For Chinese and mixed ethnic groups the odds ratio was not statistically significantly different from one, perhaps due to sample size.

Impact of comorbidities upon COVID-19 deaths

Deaths related to COVID-19 reflect broad underlying patterns, with more reported in men (61·3%, n = 6,342) and older people (at week 15, 87% (n = 8,985) of deaths were in those aged over 65, 69% (n = 7,135) were in people aged over 75). Data from the ONS across 2019 show an increased proportion of health conditions (chest and breathing issues and heart/blood pressure/circulatory problems*)* related to age. The percentages of people with heart, blood pressure, or circulatory problems were 0.48% (16-19 years); 6.31% (20-39 years); 31.35% (40-59 years); and 61.86% (60+ years). Similarly, the percentages of people with chest and breathing problems was 4.85% (16-19 years); 26.96% (20-39 years); 32.23% (40-59 years); and 35.96%

[i] Note caution must be exercised in interpreting the values in Table 1 as they do not contain measures of uncertainty

For peer review only

(60+ years)[ii]. Long-term comorbidities such as ischaemic heart disease and hypertensive disease are commonly present in men dying with COVID-19 (Figure 5a), particularly in higher age groups; a similar pattern was observed for cerebrovascular diseases in women (Figure 5b). As people reach very advanced age, for both sexes the predominant comorbidities are dementia and Alzheimer's (Figures 5a and 5b).

DISCUSSION

This analysis characterises the COVID-19 pandemic in England and Wales in the context of excess death over time, by region, and risk factor. Increases in mortality in April were predominantly driven by COVID-19, but non-COVID-19 excess death also increased in April-May 2020 across all regions. Compared to historical rates of death amongst occupational groups, COVID-19 related deaths generally followed normal patterns, excepting individuals among caring, leisure, and other service occupations who were more likely to die from COVID-related illness than die from any illness. Rates of death from COVID-19 related illness are higher among Black and Ethnic Minority (BAME) populations, but small sample sizes preclude all-cause mortality comparisons. Finally, pre-existing comorbidities are a strong risk factor for COVID-19 related death and are more common among men and the elderly, explaining why these groups appear to be at excess risk of death related to COVID-19. Thus, patterns of death and excess death from COVID-19 mirror historical trends in mortality. This contextualization of COVID-19 is critical to inform plans to protect the vulnerable while helping low risk populations in society to resume more normal lifestyle patterns.

Following a period of excess deaths, in week 25 of 2020, for the first time there were fewer deaths than the equivalent previous 5 year average (65 fewer deaths), and similarly in weeks 26 through to 28 there were 917 fewer deaths than the total of the averages for those weeks in the previous five years. This is suggestive of a mortality displacement ('harvesting') impact; a proportion of the population who died at the epidemic peak (weeks 13 to 18) may have died in the shorter-term in other circumstances. This phenomenon is previously described e.g. due to impacts of heatwaves and cold spells[8] and influenza in 1918/19 (compared to deaths from tuberculosis).[9]

[ii] https://www.ons.gov.uk/peoplepopulationandcommunity/healthandsocialcare/conditionsanddiseases/adhocs/11478peoplewithlongtermhealthconditionsukjanuarytodecember2019

For peer review only

Context to age and gender

Much age and gender-related health risk is more appropriately attributable to increased prevalence of underlying comorbidities. We are more likely to die as we age, with 84% of deaths in people over 65 years, and 66% in those over 75 years.[10] Men also die earlier in most age groups and have lower life expectancies (79·2 years) than women (82·9 years)[11]. As we age our likelihood of having long-term illness increases as has been discussed. Though the burden of risk from COVID-19 lies with older age groups, more thorough epidemiological analysis may identify some subpopulations that could be classified as lower (or higher) risk. Such analysis would inform better risk management strategies, allowing mobility and economic activity amongst some low risk older populations, as well as intrinsically low risk groups such as young people.

Context to comorbidities

Patterns of co-morbidities for COVID-19-related deaths mirror the increase in these diseases with age (in non-COVID circumstances) e.g. ischaemic heart disease is more frequently experienced with age by men than women.[12] It is unclear whether an increasing representation of dementia and Alzheimer's as comorbidities is seen because they are genuine comorbidities in their own right or due to data biases. The most important comorbidities alongside dementia and Alzheimer's are chronic lower respiratory disease in females and ischaemic heart disease in males. A role for specific genes linked to dementia and Alzheimer's and poor COVID-19 response has recently been suggested[7] and warrants further investigation. It has not been possible to locate for 2020 to date the numbers of deaths by each comorbidity in its own right: these would be useful for comparison and establishment of any excess, but whether an excess of deaths with COVID-19 by any of the comorbidities will occur is unclear at the time of writing and this requires ongoing observation.

Context to ethnicity and occupation

Ethnicity and occupation are common risk factors for morbidity and mortality from infectious disease, but are not often reported in surveillance data[13]. Heightened reported risks among specific ethnic and occupational groups are alarming, and COVID-19 has brought renewed attention to health disparities inherent in the UK population, but excepting care, service, and leisure workers, the precise nature and drivers of excess COVID-19 risk in different groups remain unclear. When considering occupational risk for example, age-standardised mortality ratios (ASMRs) in different occupational categories for COVID-19 mortality must be considered alongside ASMRs for all-cause mortality. For example, when the COVID-19-associated ASMR in

For peer review only

an occupational category is high relative to deaths from all causes, this suggests COVID-19-associated impacts should be considered in managing return to work.

Consequences of COVID-19 and our response to its presence

The response to COVID-19 appears to indirectly increase non-COVID-19 mortality by reducing healthcare-seeking behaviour. A big reduction in the number of visits to accident and emergency (from 120,356 in the week commencing 16th March to 89,584 in the week commencing 23rd March)[14,15] coincides with the increase of both COVID-19 and non-COVID-19-related UK deaths . Such a drop in consultations is inconsistent with the pattern observed in the previous year, but it suggests a reluctance or inability of the public to access healthcare during lockdown. Most recently, Cancer Research UK have estimated that for every week that routine screening is paused, 7,000 people miss referrals for further tests, and 380 cancers are not diagnosed using routine screening programmes[16]: they additionally estimate that 290,000 people fewer than usual have been referred for further tests. The reasons for this may be multi-factorial reflecting reluctance, fear of the virus, and logistical difficulties for GPs. This pattern of reduced healthcare uptake foreshadows an increased health burden as routine preventive screenings, cancer treatments, dental visits, and vaccinations have been paused to some extent during the lockdown. Evidence to this effect is provided in a report to the NHS by Medefer and reported in the Times (10th May 2020) that suggests that by October 2020 approximately 7.2 million people will be on NHS waiting lists. The report estimates that 1.3 million people may already have been added to an lengthy waiting list which stood at 4.4 million people in February 2020, as a result of the combination of delays introduced into the system by various aspects of the response of both the health services and by individuals to the COVID-19 situation. However, in the immediate future a dip in mortality is occurring, compared to baseline. In Wales, where the median age is higher than in any other UK nation or region of England,[17] the rate of death per million returned to at, or below, historical levels before any other region in England. This suggests that for high risk populations (e.g. the elderly), deaths have been compressed within the time window of the pandemic. This phenomenon was previously observed among tuberculosis patients in the months and years following the 1918 Spanish Flu[9]. Thus, continued contextualization of deaths is critical to accurately assess the long-term impact of COVID-19 on health in the UK - volatility of demand should be considered in resource planning.

Solutions: Role of surveillance, need for better data reporting

What tools do we have to look at whether changes in illness patterns might be helpful in planning a response to an emerging situation such as COVID-19? ONS data are among the most

For peer review only

accurate but have limited usefulness for real-time analysis. It is crucial that information from multiple sources is synthesised and scrutinised simultaneously, balancing timeliness against accuracy. Many readily available sources can be used in combination to inform the evidence base. In other illnesses such as influenza,[18] a primary circulation in children may precede a secondary epidemic in the wider population. Of relevance to COVID-19 is syndromic surveillance reporting, which illustrated a spike in consultations for influenza-like illness in the under 15s above baseline for weeks 49 to 51 of 2019[19]. This, considered in tandem with other syndromic surveillance data , which indicated increased trips for influenza-like illness to accident and emergency in the same period[14], have had the potential to alert society to anomalies earlier than the documented timescale for the COVID-19 pandemic. Combined scrutiny of such sources is useful to identify anomalous patterns, triggering a public health response. For example, coincident with the first reported case of COVID-19 in the UK, calls reporting cough or cold/flu and diarrhea spiked, then fell when the NHS 111 changed their call triage system[19]. Ensuring the comparability of age categories across reporting systems, and reporting data openly at the highest resolution which respects patient anonymity, aids rapid responsive production of research.

Conclusions

Policy makers have relied on models in the early phase of COVID-19. These must be supported by data-driven evidence on when, where, who and why people get sick and die. Timely emergence and analysis of this information should be used to calibrate social, cultural and economic assessments of the impact of COVID-19 versus our actions to control it, if we are to return to a cautious normality.

To our knowledge this is the first study to consider reported numbers of COVID-19 illnesses and deaths in England and Wales against their historical disease context. Our research identifies and combines important, open-access data to inform a more nuanced response to emerging disease. Many openly available resources could improve response planning for emerging disease situations such as COVID-19, and could be used to anticipate wider consequences than immediate infection-related impacts and plan accordingly. Syndromic surveillance data combined with real-time surveillance data would supplement and strengthen the mathematical models informing emerging disease responses. Our analysis highlights the importance of

For peer review only

calibrating social, cultural and economic assessments of the direct impact of COVID-19 against potential control actions.

Data sharing statement

Data and reports consulted in this study are in the public domain. The principal data source was the Office for National Statistics (ONS), which provides gold-standard confirmed causes of death for UK residents; dashboards from Public Health England (PHE) and syndromic surveillance in England via NHS 111 were additionally consulted. Use of ONS data is licensed under the Open Government Licence v.3.0, and data are available from https://www.ons.gov.uk/; Public Health England dashboard is located at https://coronavirus.data.gov.uk/; and NHS 111 reports are available via https://digital.nhs.uk.

Contributorship statement

All authors source data and contributed to analysis, and collaborated on the manuscript.

Conflict of interest statement

There are no conflicts of interest.

Ethics statement

Ethical approval was not required for this study as it is an analysis of public domain data.

Patient and Public Involvement statement

No patients involved.

References

1 Lan F-Y, Wei C-F, Hsu Y-T, Christiani DC, Kales SN. Work-related Covid-19 transmission. *MedRxiv* 2020; **286**.

2 van Doremalen N, Bushmaker T, Morris DH, *et al.* Aerosol and Surface Stability of SARS-CoV-2 as Compared with SARS-CoV-1. *N Engl J Med* 2020; **382**: 1564–7.

3 The OpenSAFELY Collaborative, Williamson E, Walker AJ, *et al.* OpenSAFELY: factors

For peer review only

associated with COVID-19-related hospital death in the linked electronic health records of 17 million adult NHS patients. *medRxiv* 2020. DOI:https://doi.org/10.1101/2020.05.06.20092999.

4 Banerjee A, Pasea L, Harris S, *et al.* Estimating excess 1- year mortality from COVID-19 according to underlying conditions and age in England: a rapid analysis using NHS health records in 3.8 million adults. *medRxiv* 2020. DOI:10.1101/2020.03.22.20040287.

5 ONS. Coronavirus (COVID-19) related deaths by ethnic group, England and Wales: 2 March 2020 to 10 April 2020. 2020. https://www.ons.gov.uk/peoplepopulationandcommunity/birthsdeathsandmarriages/deaths/articles/coronavirusrelateddeathsbyethnicgroupenglandandwales/2march2020to10april2020.

6 McIntyre M, Thaxton G, Clough H, Harris J, Rushton J. Coronavirus: patterns of illness and death mainly mirror what was already happening before the pandemic. Conversat. 2020. https://theconversation.com/coronavirus-patterns-of-illness-and-death-mainly-mirror-what-was-already-happening-before-the-pandemic-136572 (accessed May 11, 2020).

7 Kuo C-L, Pilling LC, Atkins JL, *et al.* APOE E4 GENOTYPE PREDICTS SEVERE COVID-19 IN THE UK BIOBANK COMMUNITY COHORT. *medRxiv* 2020. DOI:10.1101/2020.05.07.20094409.

8 Huynen MMTE, Martens P, Schram D, Weijenberg MP, Kunst AE. The Impact of Heat Waves and Cold Spells on Mortality Rates in the Dutch Population. *Environ Health Perspect* 2001. DOI:10.2307/3454704.

9 Noymer A. The 1918 influenza pandemic hastened the decline of tuberculosis in the United States: An age, period, cohort analysis. *Vaccine* 2011. DOI:10.1016/j.vaccine.2011.02.053.

10 ONS. Deaths by single year of age tables, UK. 2020. https://www.ons.gov.uk/peoplepopulationandcommunity/birthsdeathsandmarriages/deaths/datasets/deathregistrationssummarytablesenglandandwalesdeathsbysingleyearofagetables.

11 ONS. Health state life expectancies, UK: 2016-2018. 2019. https://www.ons.gov.uk/peoplepopulationandcommunity/healthandsocialcare/healthandlifeexpectancies/bulletins/healthstatelifeexpectanciesuk/2016to2018#life-expectancy-in-the-uk.

12 Regitz-Zagrosek V, Oertelt-Prigione S, Prescott E, *et al.* Gender in cardiovascular diseases: Impact on clinical manifestations, management, and outcomes. *Eur Heart J* 2016. DOI:10.1093/eurheartj/ehv598.

For peer review only

13 Pareek M, Bangash MN, Pareek N, *et al.* Ethnicity and COVID-19: an urgent public health research priority. Lancet. 2020. DOI:10.1016/S0140-6736(20)30922-3.

14 PHE. PHE Emergency Department Syndromic Surveillance System Bulletin, week 13, 2020. 2020 https://assets.publishing.service.gov.uk/government/uploads/system/uploads/attachment_data/file/877600/EDSSSBulletin2020wk13.pdf).

15 PHE. PHE Emergency Department Syndromic Surveillance System Bulletin, week 12, 2020. 2020 https://assets.publishing.service.gov.uk/government/uploads/system/uploads/attachment_data/file/875604/EDSSSBulletin2020wk12__1_.pdf.

16 Cancer Research UK. https://www.cancerresearchuk.org/ (accessed June 2, 2020).

17 ONS. Population estimates for the UK, England and Wales, Scotland and Northern Ireland, provisional: mid-2019. 2020 https://www.ons.gov.uk/peoplepopulationandcommunity/populationandmigration/populationestimates/bulletins/annualmidyearpopulationestimates/mid2019#population-age-structures-of-uk-countries-and-english-regions.

18 Longini IM, Koopman JS, Monto AS, Fox JP. Estimating household and community transmission parameters for influenza. *Am J Epidemiol* 1982. DOI:10.1093/oxfordjournals.aje.a113356.

19 PHE. PHE Remote Health Advice Syndromic Surveillance System Bulletin, week 13, 2020. 2020 https://assets.publishing.service.gov.uk/government/uploads/system/uploads/attachment_data/file/877589/PHE_Remote_Health_Advice_Weekly_Bulletin_2020_Week_13.pdf.

For peer review only

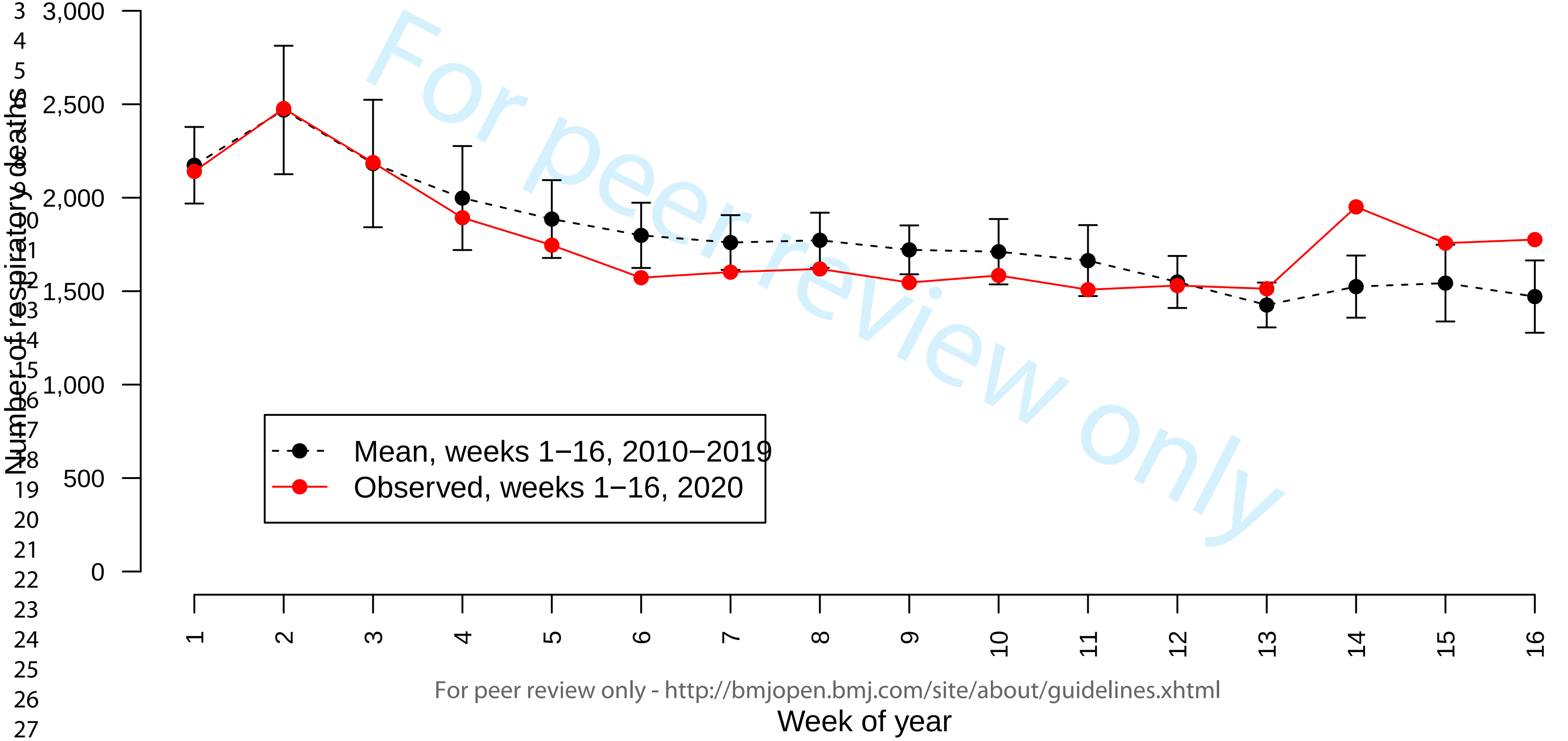

For peer review only

For peer review only - http://bmjopen.bmj.com/site/about/guidelines.xhtml

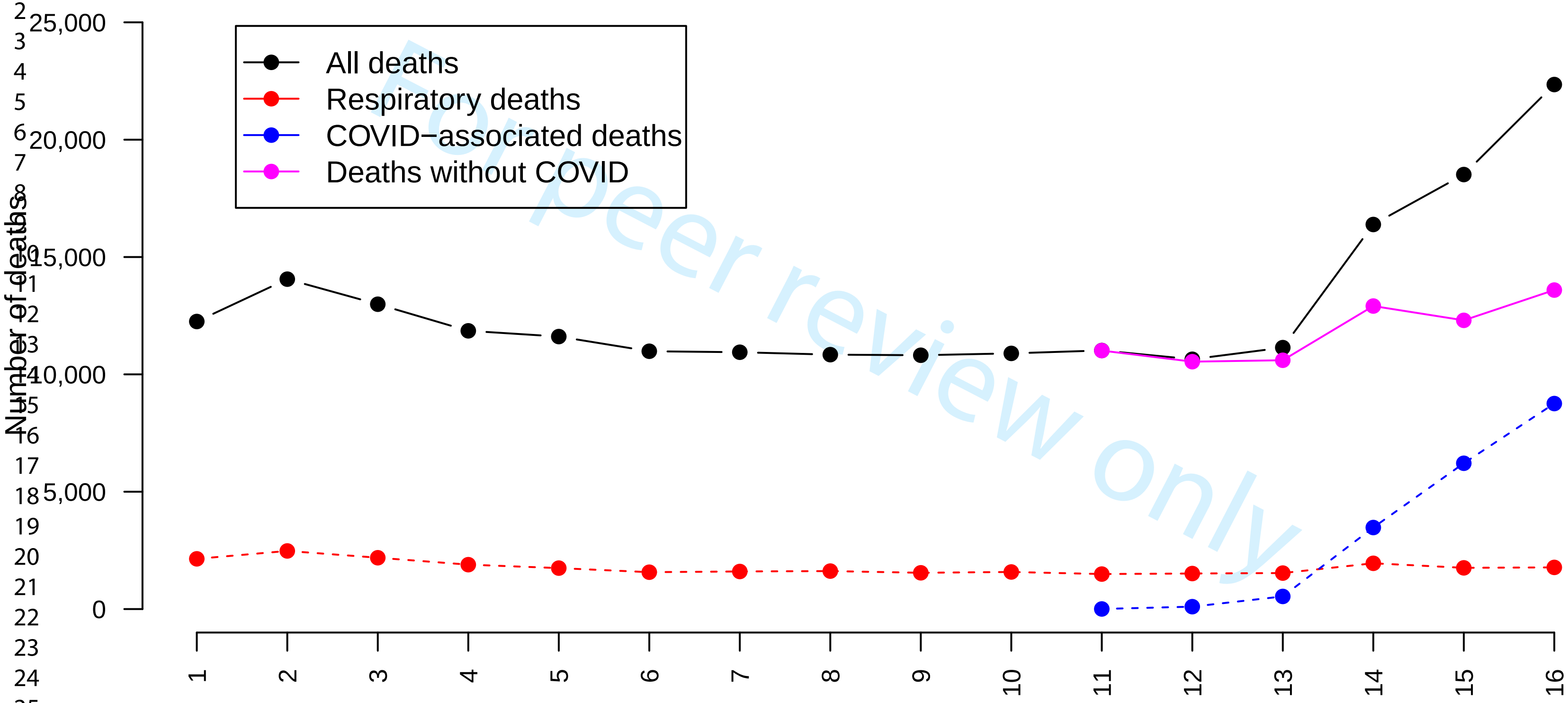

For peer review only - http://bmjopen.bmj.com/site/about/guidelines.xhtml

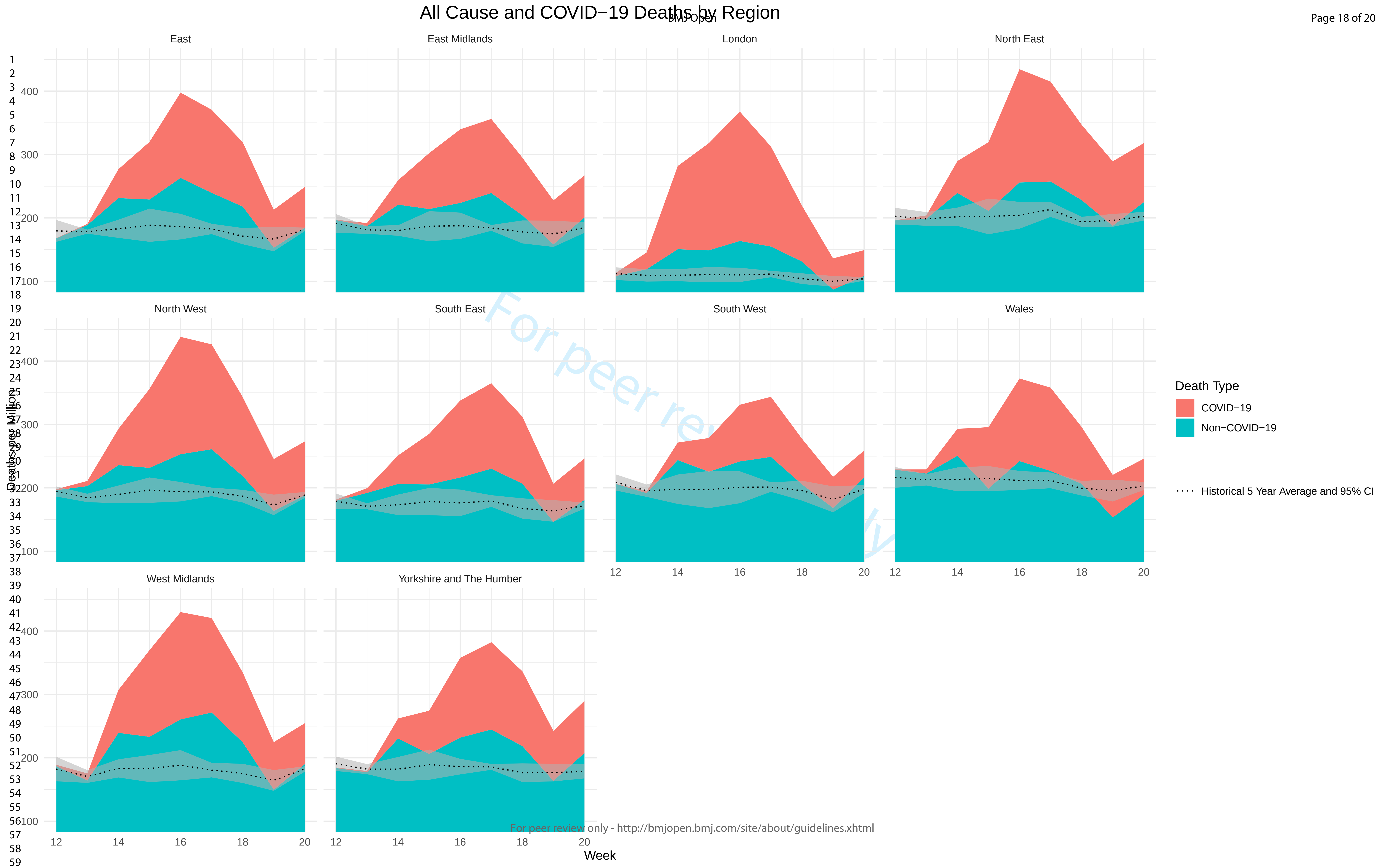
All Cause and COVID−19 Deaths by Region
East
East Midlands
London
North East
North West
South East
South West
Wales
West Midlands
Yorkshire and The Humber
Deaths per Million
Week
Death Type
COVID−19
Non−COVID−19
Historical 5 Year Average and 95% CI
For peer review only

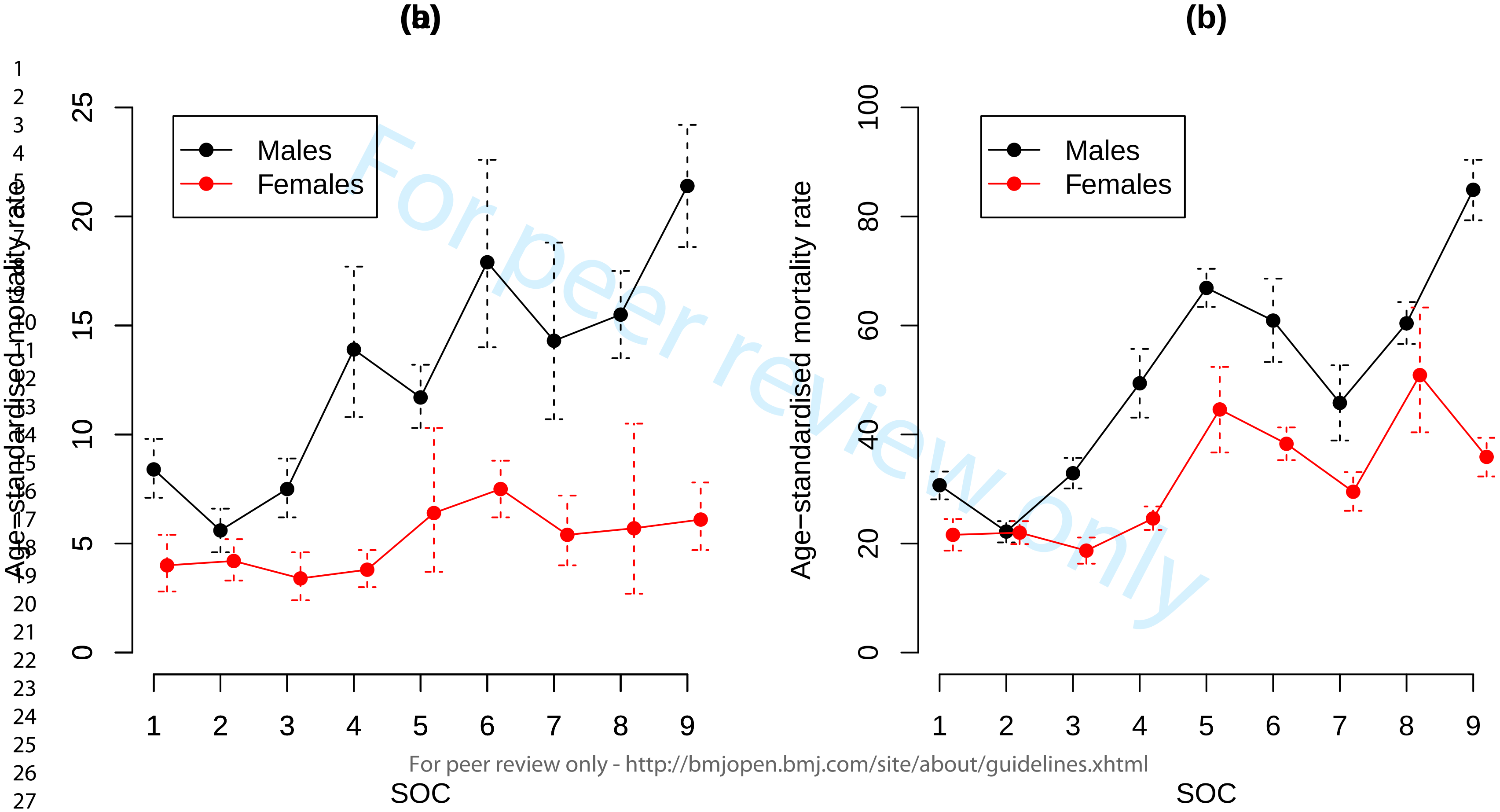

For peer review only

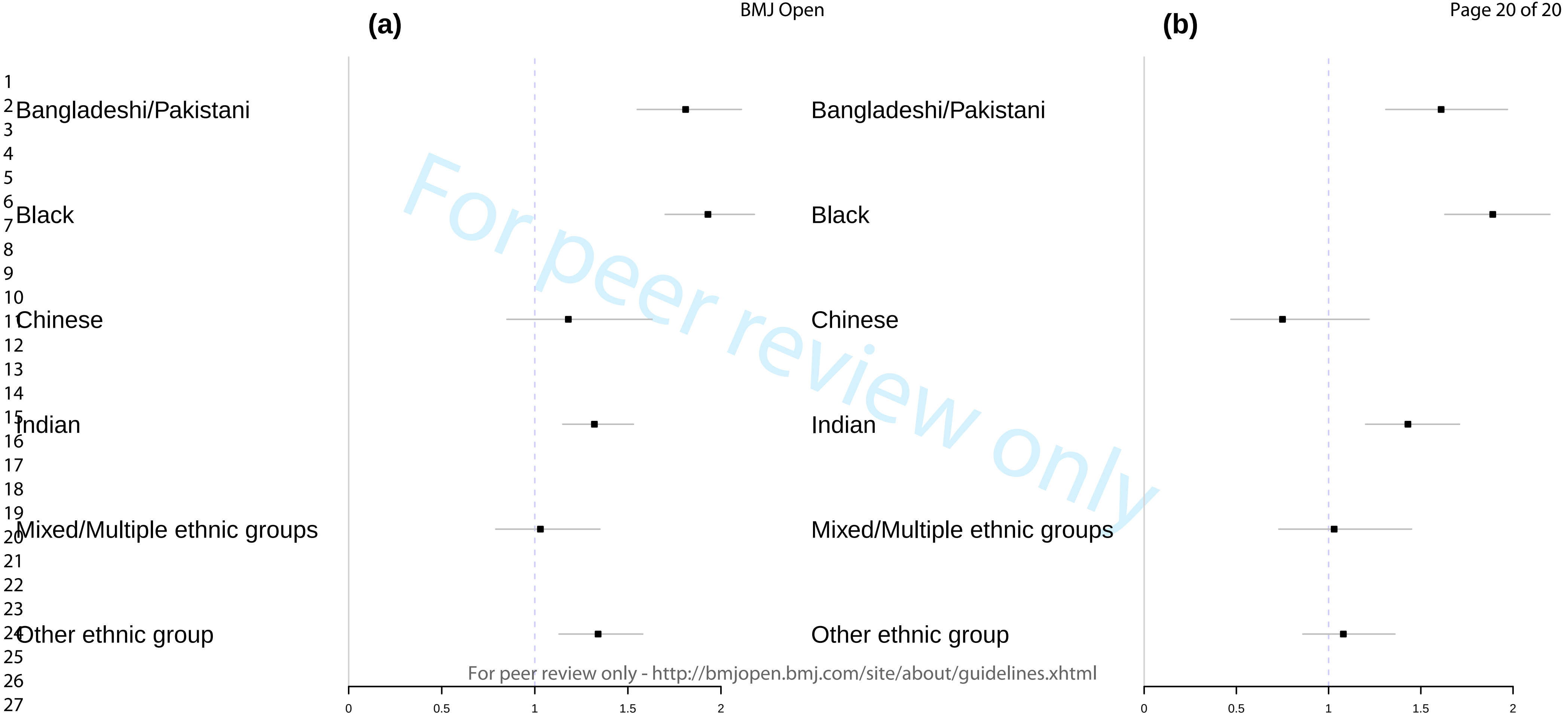

For peer review only

For peer review only - http://bmjopen.bmj.com/site/about/guidelines.xhtml

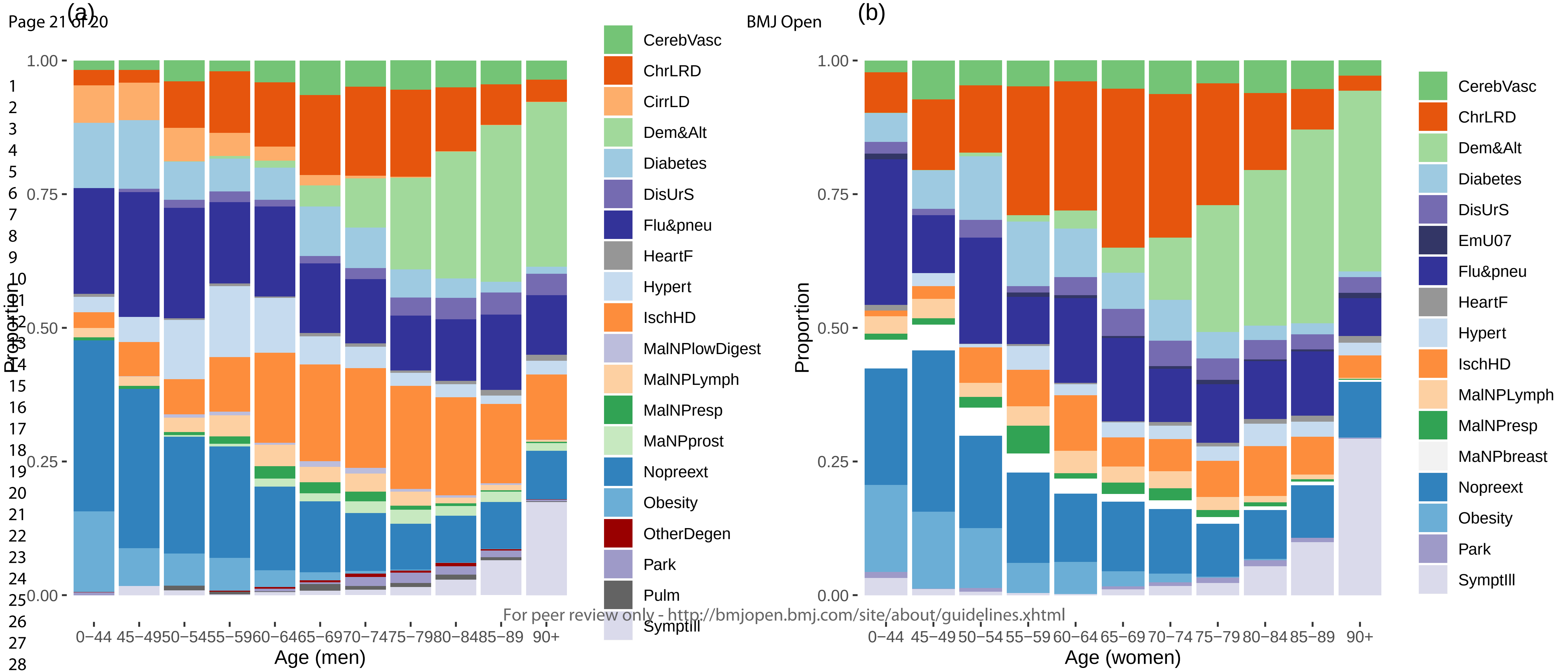

For peer review only - http://bmjopen.bmj.com/site/about/guidelines.xhtml

BMJ Open

Use of routine death and illness surveillance data to provide insight for UK pandemic planning: lessons from COVID-19

Journal:	*BMJ Open*
Manuscript ID	bmjopen-2020-044707.R1
Article Type:	Original research
Date Submitted by the Author:	17-Dec-2020
Complete List of Authors:	Clough, Helen; University of Liverpool Faculty of Health and Life Sciences, Institute of Infection, Veterinary and Ecological Sciences<br>McIntyre, Kirsty; University of Liverpool Faculty of Health and Life Sciences, Institute of Infection, Veterinary and Ecological Sciences<br>Patterson, Grace; University of Liverpool Faculty of Health and Life Sciences, Institute of Infection, Veterinary and Ecological Sciences<br>Harris, John; University of Liverpool, Institute of Population Health Sciences<br>Rushton, Jonathan; University of Liverpool Faculty of Health and Life Sciences, Institute of Infection, Veterinary and Ecological Sciences
**Primary Subject Heading**:	Epidemiology
Secondary Subject Heading:	Public health
Keywords:	STATISTICS & RESEARCH METHODS, EPIDEMIOLOGY, COVID-19, PUBLIC HEALTH

SCHOLARONE™ Manuscripts

I, the Submitting Author has the right to grant and does grant on behalf of all authors of the Work (as defined in the below author licence), an exclusive licence and/or a non-exclusive licence for contributions from authors who are: i) UK Crown employees; ii) where BMJ has agreed a CC-BY licence shall apply, and/or iii) in accordance with the terms applicable for US Federal Government officers or employees acting as part of their official duties; on a worldwide, perpetual, irrevocable, royalty-free basis to BMJ Publishing Group Ltd ("BMJ") its licensees and where the relevant Journal is co-owned by BMJ to the co-owners of the Journal, to publish the Work in this journal and any other BMJ products and to exploit all rights, as set out in our licence.

The Submitting Author accepts and understands that any supply made under these terms is made by BMJ to the Submitting Author unless you are acting as an employee on behalf of your employer or a postgraduate student of an affiliated institution which is paying any applicable article publishing charge ("APC") for Open Access articles. Where the Submitting Author wishes to make the Work available on an Open Access basis (and intends to pay the relevant APC), the terms of reuse of such Open Access shall be governed by a Creative Commons licence – details of these licences and which Creative Commons licence will apply to this Work are set out in our licence referred to above.

Other than as permitted in any relevant BMJ Author's Self Archiving Policies, I confirm this Work has not been accepted for publication elsewhere, is not being considered for publication elsewhere and does not duplicate material already published. I confirm all authors consent to publication of this Work and authorise the granting of this licence.

For peer review only

Title

Use of routine death and illness surveillance data to provide insight for UK pandemic planning: lessons from COVID-19

Authors

Corresponding author

Name: Helen E Clough

Address: Department of Livestock and One Health, Institute of Infection, Veterinary and Ecological Sciences, University of Liverpool, Leahurst Campus, Neston, Cheshire, CH64 7TE, UK.

Email: h.e.clough@liverpool.ac.uk

Other authors

Name: K Marie McIntyre

Address: Department of Livestock and One Health, Institute of Infection, Veterinary and Ecological Sciences, University of Liverpool, Leahurst Campus, Neston, Cheshire, CH64 7TE, UK.

Name: Grace E Patterson

Address: Department of Livestock and One Health, Institute of Infection, Veterinary and Ecological Sciences, University of Liverpool, Leahurst Campus, Neston, Cheshire, CH64 7TE, UK.

Name: John P Harris

Address: Department of Public Health and Policy, Institute of Population Health Sciences, University of Liverpool, Liverpool, United Kingdom L69 3BX, UK.

Name: Jonathan Rushton

Address: Department of Livestock and One Health, Institute of Infection, Veterinary and Ecological Sciences, University of Liverpool, IC2 Building, 146 Brownlow Hill, Liverpool, L3 5RF, UK.

ABSTRACT

Objectives

For peer review only

Reporting of COVID-19 cases, deaths, and testing has often lacked context for appropriate assessment of disease burden within risk groups. The research considers how routine surveillance data might provide initial insights and identify risk factors, setting COVID-19 deaths early in the pandemic into context. This will facilitate the understanding of wider consequences of a pandemic from the earliest stage, reducing fear, aiding in accurately assessing disease burden and in ensuring appropriate disease mitigation.

Setting

United Kingdom, 2020.

Participants

The study is a secondary analysis of routine, public-domain, surveillance data and information from Office for National Statistics (ONS), NHS111 and Public Health England (PHE) on deaths and disease.

Primary and secondary outcome measures

Our principal focus is ONS data on deaths mentioning COVID-19 on the death certificate. We also consider information provided in NHS111 and PHE data summaries.

Results

Deaths with COVID-19 significantly contributed to, yet do not entirely explain, abnormally elevated all-cause mortality in the UK from weeks 12-18 of 2020. Early in the UK epidemic COVID-19 was the greatest threat to those with underlying illness, rarely endangering people aged under 40 years. COVID-19 related death rates differed by region, possibly reflecting underlying population structure. Risk of COVID-19-related death was greater for health and social care staff and BAME individuals, having allowed for documented risk factors.

Conclusion

Early contextualisation of public health data is critical to recognising who gets sick, when, and why. Understanding at-risk groups facilitates a targeted response considering indirect consequences of society's reaction to a pandemic alongside disease-related impacts. COVID-19-related deaths mainly mirror historical patterns, and excess non-COVID-19 related deaths partly reflect reduced access to and uptake of healthcare during lockdown. Future outbreak response

For peer review only

will improve through better understanding of connectivity between disease monitoring systems to aid interpretation of disease risk patterns, facilitating nuanced mitigation measures.

Article summary

Strengths and limitations

- The study shows how routine, public domain data can be used to provide pertinent insight into a pandemic in its earliest stages
- The use of imaginative approaches to graphical display and numerical commentary ensures that the work can be understood by readers without a statistical specialism
- This study uses a freely available statistics package to explore public domain data sets, ensuring that results are both transparent and repeatable
- Insight is limited by problems in identifying raw data from some sources: improving ease of access will strengthen this process and improve the relevance of future inferences
- Inference is currently restricted to the UK but the same process could be applied in other countries.

Keywords

Statistics and research methods; epidemiology; COVID-19; public health

For peer review only

INTRODUCTION

Intense media reporting during the COVID-19 pandemic has focused on presenting daily data on cases, deaths and testing associated with the virus. The pandemic has undoubtedly changed our world - governments have employed unprecedented (in our times) lockdown methods to reduce transmission. These measures have greatly impacted society. The regular reporting of daily COVID-19 infections and deaths has alarmed the public, particularly when understanding of risk factors dictating severity of COVID-19 symptoms is only slowly emerging. The global population is immunologically naïve to this emerging pathogen, and society, at the time, had no available specific mitigation measures including immunological therapies, other than hand washing, social distancing, mask wearing and isolation when ill. Clinical and support staff in hospitals, health and social care staff in care homes and other settings, and key workers in transport and infrastructure industries were at increased risk of contracting the disease[1] due to frequent contact with people with high viral loads and the high aerosolised and fomite transmission potential of this virus[2]. Early analyses in England and Wales identified main risk factors for death from COVID-19 including older age, deprivation, and comorbidities[3], but did not consider how this risk differed from ‘typical’ all-cause mortality among these groups. This baseline comparison is vital to understanding what additional risk is posed by COVID-19, and to whom. Excess mortality from COVID-19 in the UK has been modelled, controlling for underlying conditions and age[4], and some conditions such as uncontrolled diabetes and severe asthma are associated with death[3]. However, understanding of the health loss impacts of COVID-19 is still limited by a lack of contextualising information, reducing our ability to respond to the challenges this disease poses, both directly and indirectly, in a proportionate, targeted manner.

We provide context for deaths and disease from COVID-19, by comparing these against a historical benchmark of when, who, and how people become ill and died pre-COVID-19. Examining associations between poor COVID-19 outcomes, demographic and socio-economic differences, age, sex and comorbidities in the context of ‘usual’ population health structures, enhances understanding of specific risk groups and hence has a role to play in maximising the effectiveness of risk mitigation strategies whilst minimising the likelihood of unnecessary and undesirable impacts. Examining excess deaths (above that normally expected at a time-point) is, furthermore, important for interpreting the total impact of a pandemic. Syndromic surveillance describing clinical symptoms and healthcare use is scrutinised to supplement clinical surveillance information used to populate the COVID-19 epidemic curve.

For peer review only

METHODS

Data sources

The principal data source was the Office for National Statistics (ONS); dashboards from Public Health England (PHE) and syndromic surveillance in England via NHS 111 were additionally consulted. Primary focus for the analyses presented was the ONS data, which provide gold-standard confirmed recorded causes of death for UK residents. Use of ONS data is licensed under the Open Government Licence v.3.0.

Statistical methods

All data must be viewed in their proper context before patterns can be inferred, and in this setting, against a historical baseline. In each case, profiles for COVID-19 deaths were considered against systematic differences in historical disease rates from appropriate comparison populations, to identify when disease was in excess of expected rates.

Causes of death were defined using the International Classification of Diseases, 10th Revision (ICD-10)[5]. Deaths involving COVID-19 were defined as those with an underlying cause, or any mention, of ICD-10 codes U07.1 (COVID-19, virus identified) or U07.2 (COVID-19, virus not identified) on the death certificate. All causes of death is the total number of deaths registered during the same time period including those involving COVID-19.

The baseline comparison group to examine weekly temporal variation in COVID-19 deaths was deaths from respiratory disease across a historical five-year period (2015 to 2019 inclusive). The mean number of respiratory deaths in weeks 1 to 16 of the year, together with an empirical 95% confidence interval, was calculated and plotted against the numbers of COVID deaths across this same time period in 2020.

When considering data regionally, rates of death per million population was the primary focus: this allowed for different population sizes within regions and hence created a metric which is comparable across geographies. Here, the mean number of deaths per million population across the previous 5-year period was used as the baseline comparison. Deaths associated with COVID-19 and excess deaths (deaths which do not attribute COVID-19 on the death certificate) were both reported.

For peer review only

Rates were again used to compare the risks associated with different Standard Occupational Categories (SOCs) for individuals between 20 and 64 years of age. Age-standardised rates per 100,000 population, standardised to the 2013 European Standard Population, were used in each category to correct for different numbers of people from different age groups working in each group, to ensure comparability between groups. Again the focus is upon the early part of the pandemic, with deaths registered up to and including 20th April 2020 constituting the data. Comparison with deaths from all causes occurring in these categories within the same timeframe creates a natural baseline for deciding how the rate of people dying with COVID-19 in a certain SOC compares with the rate of death in general in that SOC, and helps to distinguish specific COVID-related effects from more subtle societal impacts which might be influencing death rates more generally. Empirical 95% confidence intervals were provided to facilitate comparisons.

To examine the effects in black and minority ethnic (BAME) groups, again early pandemic data from 2nd March to 10th April 2020 inclusive were considered. Odds ratios are used to compare categories; these were calculated by the ONS using logistic regression models which correct for age (in 5 year categories); rural or urban inhabitants; IMD decile; socio-economic status; and self-reported health and activity status. Forest plots were used to show the estimated odds ratios for dying in each category; 95% confidence intervals were also represented.

Finally, the representation of co-morbidities amongst COVID-19 deaths in March and April 2020 was explored graphically using a stacked bar chart of the proportion of males and females separately reporting each co-morbidity across age categories (including 0-44 years; 45 to 49 years; 50 to 54 years; 55 to 59 years; 60 to 64 years; 65 to 69 years; 70 to 74 years; 75 to 79 years; 80 to 84 years; 85 to 89 years; and 90 years plus). This allows immediate comparison of how the profile of co-morbidities changes in general by age; whether different comorbidities are more readily apparent in males and females; and whether the evolution of comorbidities as age increases, differs for the two sexes.

All statistical analyses were conducted in Microsoft Excel and the R statistical software package (http://www.r-project.org)[6], making use of the graphics package ggplot2[7].

For peer review only

RESULTS

Temporal variation in COVID-19 deaths

The number of deaths from all causes varies annually and seasonally, peaking in winter. Typically, respiratory deaths range from 10% to 22% of all deaths and are seasonal, peaking annually in January; the 2015 peak was high (16,237 deaths in week two compared to an average of 12,277 deaths that week over the previous five years) due to a severe influenza season, and 2018 similarly had a severe influenza season resulting in a high death count. The minimum number of weekly deaths over this five year period was 6,606 (week 54, 2013). In 2020 deaths from respiratory infections were lower than the mean in the previous five years until early April (week 14), after which they became higher than historical rates when including deaths from COVID-19 (Figure 1a) (in week 14 observed respiratory deaths exceeded the 5-year historical upper 95% confidence interval limit by 146). An excess of unexplained deaths becomes clear from week 14 onwards (Figure 1b). Following a period of excess deaths, in week 25 of 2020, for the first time there were fewer deaths than the equivalent previous 5 year average (65 fewer deaths), and similarly in weeks 26 through to 28 there were 917 fewer deaths than the total of the averages across years for those weeks in the previous five years[8].

FIGURE 1a AND 1b HERE

Regional differences in COVID-19 deaths

Regions of England and Wales experience different death rates and this pattern is true for deaths from COVID-19 (Figure 2) e.g. rates were highest and peaked in week 17 in London (204 per million), the North-West (185 per million), the North-East (179 per million), and the West Midlands (169 per million). Peak rates were lowest in the South-West (95 per million) and East Midlands (116 per million). From weeks 13-18 (March 23-May 3), all regions of England and Wales experienced excess non-COVID-19 related deaths. This was most apparent in the West Midlands in week 17 (starting April 20), with a peak of approximately 91 deaths per million. Between weeks 13-18 (March 23- May 3) there were 46,594 excess deaths in England and Wales, 13,399 of which were listed as non-COVID-19-related. In week 25 (19th June) the total deaths dipped below the 5-year historical average for the first time (9,339 compared to 9,404) and this pattern continued until 10th July.

FIGURE 2 HERE.

For peer review only

Occupational differences in COVID-19 deaths

After age-standardisation (rates per 100,000 population, standardised to the 2013 European Standard Population), men employed in lower skilled occupations (21.4, 95% CI 18.6-24.2) (Figure 3a) were more likely to die of COVID-related illness (k=225 deaths from n=1321 deaths in total across occupations for men[9]), as was true for all-cause mortality (Figure 3b, k=915 deaths out of n=5627 deaths). This differs for women, where those employed as carers in health and social care, leisure and other service operations (Figure 3a) were most likely to die from COVID-related illness (k=130 deaths out of n=531 deaths in total across occupations for women), but not more likely to die if examining all-cause mortality (Figure 3b, k=651 deaths from n=3003 deaths). For both men and women, the less technical and more manual their occupation (using ONS SOC 2020 categories), the greater the risk of dying from any cause including COVID-19-related disease. In addition, when occupations are more manual, variation in age-standardised mortality rates is higher, particularly for men or women in certain SOC categories e.g. men undertaking administrative and secretarial roles; women in skilled trades; men in caring, leisure and other service occupations; men in sales and customer service roles; women working as process, plant and machine operatives; and men undertaking low skilled elementary roles.

FIGURE 3a AND 3b HERE.

A crude comparison suggests that age-standardised mortality rates for most occupations are reduced by COVID-19 relative to deaths from any cause (Table 1), with the rate only increased (for both sexes) in caring, leisure and other service occupations.

Table 1: Ratio of estimated age-standardised mortality rates comparing occupational categories with a baseline of managerial workers (SOC group 1) for (a) COVID-19 associated male and female deaths; and (b) all-cause male and female deaths (including COVID-19). Note caution must be exercised in interpreting the values in Table 1 as they do not contain measures of uncertainty

SOC	Group	Females		Males	
		COVID	All	COVID	All

For peer review only

1	Managers, directors and senior officials (baseline)	-	-	-	-
2	Professional occupations	1.05	1.02	0.67	0.72
3	Associate professional & technical occupations	0.85	0.87	0.89	1.07
4	Administrative & secretarial occupations	0.95	1.14	1.65	1.61
5	Skilled trades occupations	1.60	2.06	1.39	2.18
6	Caring, leisure & other service occupations	1.88	1.77	2.13	1.98
7	Sales & customer service occupations	1.35	1.37	1.70	1.49
8	Process, plant & machine operatives	1.43	2.36	1.85	1.97
9	Low skilled elementary occupations	1.53	1.66	2.55	2.77

Ethnic associations with COVID-19 deaths

As previously reported by the ONS[10] in data from 2nd March to 10th April 2020, there were increased odds of dying from COVID-19 for Bangladeshi/Pakistani (386 deaths), Black (766 deaths) and Indian (483 deaths) ethnic groups (for both sexes) when compared with a baseline white group and adjusted for age, region, rural/urban, Index of Multiple Deprivation decile, household composition, socio-economic status and underlying health Conditions (Figure 4a and 4b). In total across all groups, in this time period 12,805 deaths occurred. For Chinese and mixed ethnic groups the odds ratio was not statistically significantly different from one, perhaps due to small sample size (59 deaths in total observed in Chinese ethnic groups in this time period, from a total of 12,805 across all categories).

FIGURE 4a AND 4b HERE.

Impact of comorbidities upon COVID-19 deaths

Deaths related to COVID-19 reflect broad underlying patterns, with more reported in men (61.3%, n=6,342) and older people (at week 15, 87% (n=8,985) of deaths were in those aged over 65, 69% (n=7,135) were in people aged over 75). Data from the ONS across 2019 show an increased proportion of health conditions (chest and breathing issues and heart/blood pressure/circulatory problems*)* related to age. The percentages of people with heart, blood pressure, or circulatory problems were 0.48% (16-19 years); 6.31% (20-39 years); 31.35% (40-59 years); and 61.86% (60+ years). Similarly, the percentages of people with chest and breathing problems were 4.85% (16-19 years); 26.96% (20-39 years); 32.23% (40-59 years); and 35.96% (60+ years)[11]. Long-term comorbidities such as ischaemic heart disease and hypertensive disease are commonly present in men dying with COVID-19 (Figure 5a), particularly in higher age groups; a similar pattern was observed for cerebrovascular diseases in women (Figure 5b).

For peer review only

As people reach very advanced age, for both sexes the predominant comorbidities are dementia and Alzheimer's (Figures 5a and 5b).

FIGURE 5a AND 5b HERE.

Impacts of our response to COVID-19

Numerous other resources can provide information about the impacts of the human response to the pandemic. The response to COVID-19 appears to indirectly increase non-COVID-19 mortality by reducing healthcare-seeking behaviour: a big reduction in the number of visits to accident and emergency units (from 120,356 in the week commencing 16th March to 89,584 in the week commencing 23rd March)[12,13] coincides with the increase of both COVID-19 and non-COVID-19-related UK deaths. There are wide impacts on a range of non-communicable diseases: for example, Cancer Research UK have estimated that for every week that routine screening is paused, 7,000 people miss referrals for further tests, and 380 cancers are not diagnosed using routine screening programmes[14]: they additionally estimate that 290,000 people fewer than usual have been referred for further tests.

Data suggests that routine preventive screenings, cancer treatments, dental visits, and vaccinations have all been paused to some extent during the lockdown. Evidence for this is provided in a report to the NHS by Medefer, reported in the Times (10th May 2020). It suggests that by October 2020, approximately 7.2 million people will be on NHS waiting lists. The report estimates that 1.3 million people may already have been added to a lengthy waiting list which stood at 4.4 million people in February 2020.

DISCUSSION

This analysis characterises the COVID-19 pandemic in England and Wales in the context of excess death over time, by region, and risk factor. Increases in mortality in April were predominantly driven by COVID-19, but non-COVID-19 excess deaths also increased in April-May 2020 across all regions. Compared to historical rates of death amongst occupational groups, COVID-19 related deaths generally followed normal patterns, excepting individuals among caring, leisure, and other service occupations who were more likely to die from COVID-related illness than die from any illness. Rates of death from COVID-19 related illness are higher among Black and Ethnic Minority (BAME) populations, but small sample sizes preclude all-cause

For peer review only

mortality comparisons. Finally, pre-existing comorbidities are a strong risk factor for COVID-19 related death and are more common among men and the elderly, explaining why these groups appear to be at excess risk of death related to COVID-19. Thus, patterns of death and excess death from COVID-19 mirror historical trends in mortality. This contextualisation of COVID-19 is critical to inform plans to protect the vulnerable while helping low risk populations in society to resume more normal lifestyle patterns.

The lower-than-expected death toll from week 25 onwards may be suggestive of a mortality displacement ('harvesting') impact; a proportion of the population who died at the epidemic peak (weeks 13 to 18) may have died in the shorter-term in other circumstances. The complete picture is likely to be far more complex, but the harvesting phenomenon is previously described e.g. due to impacts of heatwaves and cold spells[15] and influenza in 1918/19 (compared to deaths from tuberculosis)[16]. Such population readjustments need to be taken into account in planning processes as the overall health loss may be relatively small compared to a disease or health problem that kills people who are healthy.

Context to age and gender

Much age and gender-related health risk is more appropriately attributable to increased prevalence of underlying comorbidities. We are more likely to die as we age, with 84% of annual deaths in people over 65 years, and 66% in those over 75 years[17]. Men also die earlier in most age groups and have lower life expectancies (79.2 years) than women (82.9 years)[18]. As we age our likelihood of having long-term illness increases as has been discussed. Though the burden of risk from COVID-19 lies with older age groups, more thorough epidemiological analysis may identify some subpopulations that could be classified as lower (or higher) risk. Such analysis would inform better risk management strategies, allowing mobility and economic activity amongst some low-risk older populations, as well as intrinsically low risk groups such as young people.

Context to comorbidities

Patterns of co-morbidities for COVID-19-related deaths mirror the increase in these diseases with age (in non-COVID circumstances) e.g. ischaemic heart disease is more frequently experienced with age by men than women[19]. It is unclear whether an increasing representation of dementia and Alzheimer's as comorbidities is seen because they are genuine comorbidities in their own right or due to data biases. The most important other comorbidities are chronic lower respiratory disease in females and ischaemic heart disease in males. A role for

For peer review only

specific genes linked to dementia and Alzheimer's and poor COVID-19 response has recently been suggested[20] and warrants further investigation. It has not been possible to locate for 2020, the numbers of deaths by each comorbidity in its own right: these would be useful for comparison and establishment of any excess, but whether an excess of deaths with COVID-19 by any of the comorbidities will occur is unclear at the time of writing.

Context to ethnicity and occupation

Ethnicity and occupation are common risk factors for morbidity and mortality from infectious disease, but are not often reported in surveillance data[21]. Heightened reported risks among specific ethnic and occupational groups are alarming, and COVID-19 has brought renewed attention to health disparities inherent in the UK population, but excepting care, service, and leisure workers, the precise nature and drivers of excess COVID-19 risk in different groups remains unclear. When considering occupational risk for example, age-standardised mortality ratios (ASMRs) in different occupational categories for COVID-19 mortality must be considered alongside ASMRs for all-cause mortality. For example, when the COVID-19-associated ASMR in an occupational category is high relative to deaths from all causes, this suggests COVID-19-associated impacts should be considered in managing return to work.

Consequences of COVID-19 and our response to its presence

The reduction in accident and emergency consultations is inconsistent with the pattern observed in 2019; it suggests a reluctance or inability of the public to access healthcare during lockdown. Unfortunately, comparisons against a longer range of historical data are not possible since the surveillance system changed in 2018, with greater numbers of hospitals reporting to the system from this point onwards. The reasons for this reduction may be multi-factorial reflecting reluctance, fear of the virus, and logistical difficulties for GPs. This pattern of reduced healthcare uptake foreshadows an increased health burden as a result of the combination of delays introduced into the system by aspects of both the health services and individuals' responses to COVID-19. However, in the immediate future, a dip in mortality is occurring, compared to baseline. In Wales, where the median age is higher than in any other UK nation or region of England[22], the rate of death per million returned to at, or below, historical levels before any other region in England. This suggests that for high-risk populations (e.g. the elderly), deaths have been compressed within the time window of the pandemic. This phenomenon was previously observed among tuberculosis patients in the months and years following the 1918 Spanish Flu[16]. Thus, continued contextualisation of deaths is critical to accurately assess the

For peer review only

long-term impact of COVID-19 on health in the UK - volatility of demand should be considered in resource planning.

Solutions: Role of surveillance, need for better data reporting

What tools do we have to look at whether changes in illness patterns might be helpful in planning a response to an emerging situation such as COVID-19? ONS data are among the most accurate but have limited usefulness for real-time analysis. It is crucial that information from multiple sources is synthesised and scrutinised simultaneously, balancing timeliness against accuracy. Many readily available sources can be used in combination to inform the evidence-base. In other illnesses such as influenza[23], a primary circulation in children may precede a secondary epidemic in the wider population. Of relevance to COVID-19 is syndromic surveillance reporting, which illustrated a spike in consultations for influenza-like illness in the under 15s above baseline for weeks 49 to 51 of 2019[24]. This, considered in tandem with other syndromic surveillance data, which indicated increased trips for influenza-like illness to accident and emergency in the same period[12], have the potential to alert society to anomalies earlier than the documented timescale for the COVID-19 pandemic. Combined scrutiny of such sources is useful to identify anomalous patterns, triggering a public health response. For example, coincident with the first reported case of COVID-19 in the UK, calls reporting cough or cold/flu and diarrhea spiked, then fell when the NHS 111 changed their call triage system[24]. Ensuring the comparability of age categories across reporting systems, and reporting data openly at the highest resolution which respects patient anonymity, aids rapid responsive production of understanding from research. On the international stage, authors in the US have identified analogous issues with non-integrated reporting systems; they developed an ‘App’ that attempts to address some of the issues[25]. In Europe, two surveillance strands are followed and both are restricted access: EU/EEA Member States and the UK report for every 24 hour period the number of laboratory-confirmed cases of COVID-19 using their Early Warning and Response System (EWRS). Enhanced surveillance has also been put in place via The European Surveillance System – TESSy[26]. The restricted access nature of these resources limits their real-time applicability for parties other than those with permitted access. A full consideration of the international picture is beyond the scope of this paper, but the process described herein could be repeated for other populations.

Solutions: A model for success

Taiwan provides perhaps the best example of success in rapidly containing and controlling COVID-19; they eliminated the virus by April 2020 without going into lockdown, with minimal

For peer review only

economic damage and few deaths[27]. Taiwan's plan for success against COVID-19 can be summarised in four points[28]. 1) In response to previous experience with SARS in 2003 and influenza H1N1 in 2009, Taiwan had developed highly functional pandemic response plans and infrastructure that were immediately operationalised in early 2019, including a Central Epidemic Command Centre and community surveillance system. 2) Taiwanese officials were quick to respond to the earliest whistle-blower reports from China with significant travel restrictions and activation of pandemic response plans. 3) The Taiwanese government is trusted and was able to successfully balance government oversight with regional autonomy. Localities and private establishments were trusted to run their own track and trace systems, which were designed to be easily linked up to provide national coverage. Privacy concerns are acknowledged and managed, but the proven results obtained drive high levels of participation. 4) High buy-in from civilians across all aspects of disease control. Civilians are given space to provide suggestions and concerns in online town-halls. Civilians are provided with adequate monetary support while quarantining, but also face large fines, leading to high compliance. While there are cultural, social, and geographic differences between the UK and Taiwan, many of these actions could be successfully deployed in the UK.

CDC specify a series of steps to be followed in investigating and responding to an outbreak Figure 6 outlines where this research contributes to that process, and how it feeds into the wider process of outbreak management. It is clear from this figure how timely data from a variety of sources, at closely aligned degrees of temporal and spatial resolution, would streamline public health processes, significantly enhancing capacity to respond to future pandemics.

FIGURE 6 HERE.

Methodological limitations

Any analysis based upon surveillance data is subject to limitations. Biases in surveillance data are well-known and well-documented[29]. Data on cases of disease is informative but can be heavily biased by who appears in the system, and why. For example, any estimate of the case fatality ratio for COVID-19 from the early part of the pandemic would potentially be over-estimated as a consequence of the likely huge under-ascertainment of disease in the early stages, when knowledge about COVID-19 was evolving, and testing was largely limited to hospital cases of disease (the most severe manifestations). It is for this reason that the research

For peer review only

in this paper has focused on data from the ONS, which records conclusive cause of death and is the most complete and accurate resource for UK deaths which should ensure that any biases of reporting are minimised.

The analysis presented here is largely descriptive, and as such it is not possible to make any statements about, for example, statistical significance of observations. This approach is deliberate: it is the authors' intention to demonstrate how a well-chosen graphical display can provide valuable insight, which can be readily interpreted by those without specialist knowledge.

Conclusions

Policy makers have relied on models in the early phase of COVID-19. These must be supported by data-driven evidence on when, where, who and why people get sick and die. Timely emergence and analysis of this information should be used to calibrate social, cultural and economic assessments of the impact of COVID-19 versus our actions to control it, if we are to return to a cautious normality.

To our knowledge this is the first study to consider reported numbers of COVID-19 illnesses and deaths in England and Wales against their historical disease context. Our research identifies and combines important, open-access data to inform a more nuanced response to emerging disease. Many openly available resources could improve response planning for emerging disease situations such as COVID-19, and could be used to anticipate wider consequences than immediate infection-related impacts. Syndromic surveillance data combined with real-time surveillance would supplement and strengthen the mathematical models informing emerging disease responses. Our analysis highlights the importance of calibrating social, cultural and economic assessments of the direct impact of COVID-19 against potential control actions.

Data sharing statement

Data and reports consulted in this study are in the public domain. The principal data source was the Office for National Statistics (ONS), which provides gold-standard confirmed causes of death for UK residents; dashboards from Public Health England (PHE) and syndromic surveillance in England via NHS 111 were additionally consulted. Use of ONS data is licensed under the Open Government Licence v.3.0, and data are available from https://www.ons.gov.uk/; Public Health

For peer review only

England dashboard is located at https://coronavirus.data.gov.uk/; and NHS 111 reports are available via https://digital.nhs.uk.

Contributorship statement

All authors (HEC, KMM, GEP, JPH, JR) contributed equally to the planning and development of the research including its conception and design, data acquisition, analysis and interpretation of data. HEC and GEP led the writing of the manuscript. Several authors wrote and implemented the R code to produce analysis and figures: HEC (Figures 1, 3, 4, 6), GEP (Figure 2) and KMM (Figure 5, 6).

Conflict of interest statement

There are no conflicts of interest.

Ethics statement

Ethical approval was not required for this study as it is an analysis of public domain data.

Patient and Public Involvement statement

No patients involved.

Funding statement

The work was funded through the employment of the authors at the University of Liverpool, UK.

References

1 Lan F-Y, Wei C-F, Hsu Y-T, *et al.* Work-related Covid-19 transmission. *MedRxiv* 2020;**286**.

2 van Doremalen N, Bushmaker T, Morris DH, *et al.* Aerosol and Surface Stability of SARS-CoV-2 as Compared with SARS-CoV-1. *N Engl J Med* 2020;**382**:1564–7. doi:10.1056/NEJMc2004973

3 The OpenSAFELY Collaborative, Williamson E, Walker AJ, *et al.* OpenSAFELY: factors associated with COVID-19-related hospital death in the linked electronic health records of 17 million adult NHS patients. *medRxiv* Published Online First: 2020. doi:https://doi.org/10.1101/2020.05.06.20092999

4 Banerjee A, Pasea L, Harris S, *et al.* Estimating excess 1- year mortality from COVID-19 according to underlying conditions and age in England: a rapid analysis using NHS health records in 3.8 million adults. *medRxiv* Published Online First: 2020.

For peer review only

doi:10.1101/2020.03.22.20040287

5 World Health Organization: International statistical classification of diseases and related health problems. 10th revision, fifth edition, 2016. ISBN 978 92 4 154916 5.

6 R Core Team. R: A language and environment for statistical computing. R Foundation for Statistical Computing. 2020.https://www.r-project.org/

7 Wickham H. *ggplot2: Elegant Graphics for Data Analysis*. New York: : Springer-Verlag 2016. https://ggplot2.tidyverse.org

8 ONS. Deaths registered weekly in England and Wales, provisional. 2020.https://www.ons.gov.uk/peoplepopulationandcommunity/birthsdeathsandmarriages/deaths/datasets/weeklyprovisionalfiguresondeathsregisteredinenglandandwales

9 ONS. Coronavirus (COVID-19) related deaths by occupation, England and Wales. 2020.https://www.ons.gov.uk/peoplepopulationandcommunity/healthandsocialcare/causesofdeath/datasets/coronaviruscovid19relateddeathsbyoccupationenglandandwales/current

10 ONS. Coronavirus (COVID-19) related deaths by ethnic group, England and Wales: 2 March 2020 to 10 April 2020. 2020.https://www.ons.gov.uk/peoplepopulationandcommunity/birthsdeathsandmarriages/deaths/articles/coronavirusrelateddeathsbyethnicgroupenglandandwales/2march2020to10april2020

11 ONS. People with long-term health conditions, UK: January to December 2019. 2020.https://www.ons.gov.uk/peoplepopulationandcommunity/healthandsocialcare/conditionsanddiseases/adhocs/11478peoplewithlongtermhealthconditionsukjanuarytodecember2019

12 PHE. PHE Emergency Department Syndromic Surveillance System Bulletin, week 13, 2020. 2020. https://assets.publishing.service.gov.uk/government/uploads/system/uploads/attachment_data/file/877600/EDSSSBulletin2020wk13.pdf)

13 PHE. PHE Emergency Department Syndromic Surveillance System Bulletin, week 12, 2020. 2020. https://assets.publishing.service.gov.uk/government/uploads/system/uploads/attachment_data/file/875604/EDSSSBulletin2020wk12__1_.pdf

14 Cancer Research UK. https://www.cancerresearchuk.org/ (accessed 2 Jun 2020).

15 Huynen MMTE, Martens P, Schram D, *et al.* The Impact of Heat Waves and Cold Spells on Mortality Rates in the Dutch Population. *Environ Health Perspect* Published Online First:

For peer review only

2001. doi:10.2307/3454704

16 Noymer A. The 1918 influenza pandemic hastened the decline of tuberculosis in the United States: An age, period, cohort analysis. *Vaccine* Published Online First: 2011. doi:10.1016/j.vaccine.2011.02.053

17 ONS. Deaths by single year of age tables, UK:2018 Edition. 2020.https://www.ons.gov.uk/peoplepopulationandcommunity/birthsdeathsandmarriages/deaths/datasets/deathregistrationssummarytablesenglandandwalesdeathsbysingleyearofagetables

18 ONS. Health state life expectancies, UK: 2016-2018. 2019.https://www.ons.gov.uk/peoplepopulationandcommunity/healthandsocialcare/healthandlifeexpectancies/bulletins/healthstatelifeexpectanciesuk/2016to2018#life-expectancy-in-the-uk

19 Regitz-Zagrosek V, Oertelt-Prigione S, Prescott E, *et al.* Gender in cardiovascular diseases: Impact on clinical manifestations, management, and outcomes. *Eur Heart J* Published Online First: 2016. doi:10.1093/eurheartj/ehv598

20 Kuo C-L, Pilling LC, Atkins JL, *et al.* APOE E4 GENOTYPE PREDICTS SEVERE COVID-19 IN THE UK BIOBANK COMMUNITY COHORT. *medRxiv* Published Online First: 2020. doi:10.1101/2020.05.07.20094409

21 Pareek M, Bangash MN, Pareek N, *et al.* Ethnicity and COVID-19: an urgent public health research priority. Lancet. 2020. doi:10.1016/S0140-6736(20)30922-3

22 ONS. Population estimates for the UK, England and Wales, Scotland and Northern Ireland, provisional: mid-2019. 2020.https://www.ons.gov.uk/peoplepopulationandcommunity/populationandmigration/populationestimates/bulletins/annualmidyearpopulationestimates/mid2019#population-age-structures-of-uk-countries-and-english-regions

23 Longini IM, Koopman JS, Monto AS, *et al.* Estimating household and community transmission parameters for influenza. *Am J Epidemiol* Published Online First: 1982. doi:10.1093/oxfordjournals.aje.a113356

24 PHE. PHE Remote Health Advice Syndromic Surveillance System Bulletin, week 13, 2020. 2020. https://assets.publishing.service.gov.uk/government/uploads/system/uploads/attachment_data/file/877589/PHE_Remote_Health_Advice_Weekly_Bulletin_2020_Week_13.pdf

25 Weingarten S, Slotkin JR, Alkire M. Building A Real-Time Covid-19 Early-Warning System. Harv. Bus. Rev. 2020.https://hbr.org/2020/06/building-a-real-time-covid-19-early-

For peer review only

warning-system

26 The European Surveillance System (TESSy). https://www.ecdc.europa.eu/en/publications-data/european-surveillance-system-tessy

27 Summers DJ, Cheng DH-Y, Lin PH-H, *et al.* Potential lessons from the Taiwan and New Zealand health responses to the COVID-19 pandemic. *Lancet Reg Heal - West Pacific* Published Online First: 2020. doi:10.1016/j.lanwpc.2020.100044

28 Su SF, Han YY. How Taiwan, a non-WHO member, takes actions in response to COVID-19. *J Glob Health* Published Online First: 2020. doi:10.7189/JOGH.10.010380

29 Haut ER, Pronovost PJ. Surveillance bias in outcomes reporting. JAMA - J. Am. Med. Assoc. 2011. doi:10.1001/jama.2011.822

Figure captions

Figure 1: Time series plots representing (a) Number of respiratory deaths per week in the first 16 weeks of 2020, by comparison with a temporally equivalent 5 year historic baseline mean (with 95% confidence intervals); and (b) Number of deaths; respiratory deaths; deaths with COVID-19 on the death certificate; and deaths without COVID-19 on the death certificate, across the first 16 weeks of 2020.

Figure 2: All-cause and COVID-19 deaths by region between weeks 12 and 20 of 2020.

Figure 3: Age-standardised mortality rate by Standard Occupational Category (SOC) for (a) deaths mentioning COVID-19 on the death certificate and (b) all deaths registered up to and including 20th April 2020.

Figure 4: Odds ratios by ethnic category for deaths between 2nd March to 10th April 2020 which mention COVID-19 on the death certificate.

Figure 5: Stacked barplot representing the main comorbidities for COVID-19 by age, for deaths occurring in March and April 2020. CerebVasc = cerebrovascular disease; ChrLRD = Chronic lower respiratory disease; CirrLD; Dem&Alt; Diabetes; DisUrS = Disease of the urinary system; Flu&pneu = Influenza and pneumona ; HeartF = Heart failure; Hypert = Hypertension; IschHD = Ischaemic heart disease; MaNPbreast = malignant neoplasm of the breast; MalNPlowDigest = malignant neoplasm of the lower digestive tract;

For peer review only

MalNPLymph = malignant neoplasm of the lymphatic system; MalNPresp malignant neoplasm of the respiratory system; MaNPprost malignant neoplasm of the prostate; Nopreext no pre-existing condition; Obesity; OtherDegen = Other degenerative disease; Park = Parkinson's disease; Pulm = Pulmonary disease; SymptIll = Ill-defined symptoms.

Figure 6: Schematic describing the role of data, analysis and information generation in an iterative approach to pandemic management and infectious disease public health.

593

For peer review only

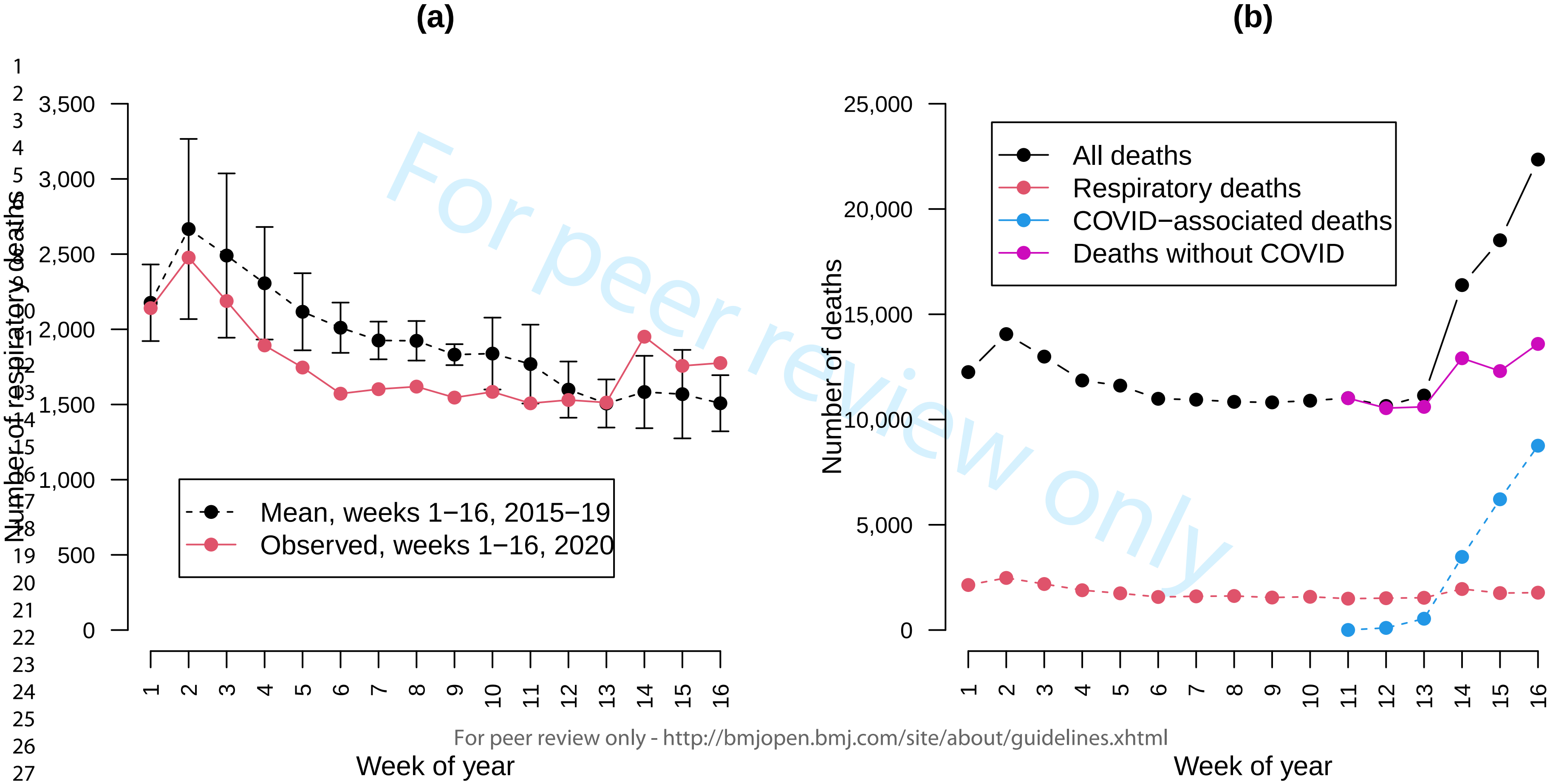

For peer review only

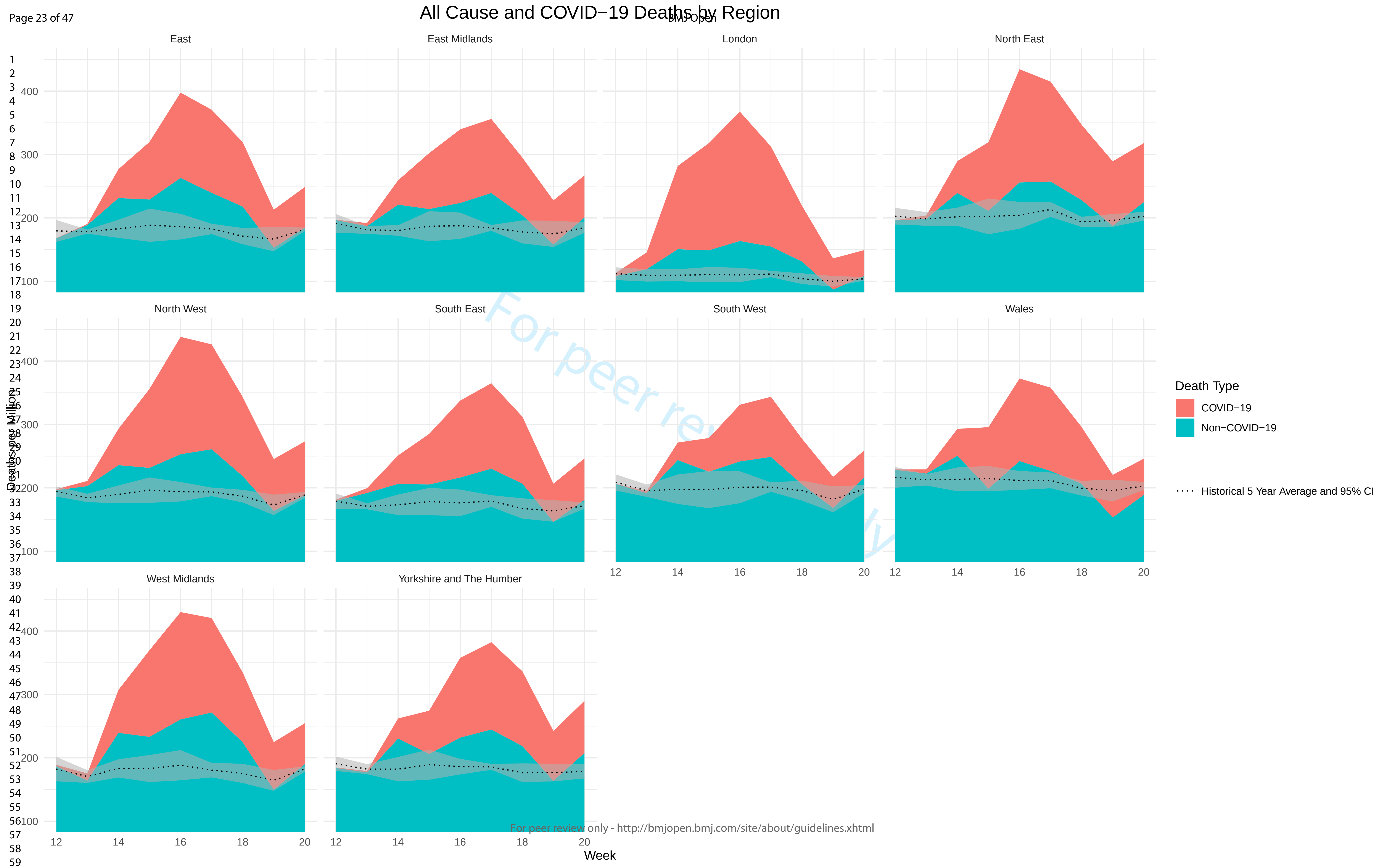
All Cause and COVID−19 Deaths by Region
East
East Midlands
London
North East
North West
South East
South West
Wales
West Midlands
Yorkshire and The Humber
Deaths per Million
Week
Death Type
COVID−19
Non−COVID−19
Historical 5 Year Average and 95% CI
For peer review only

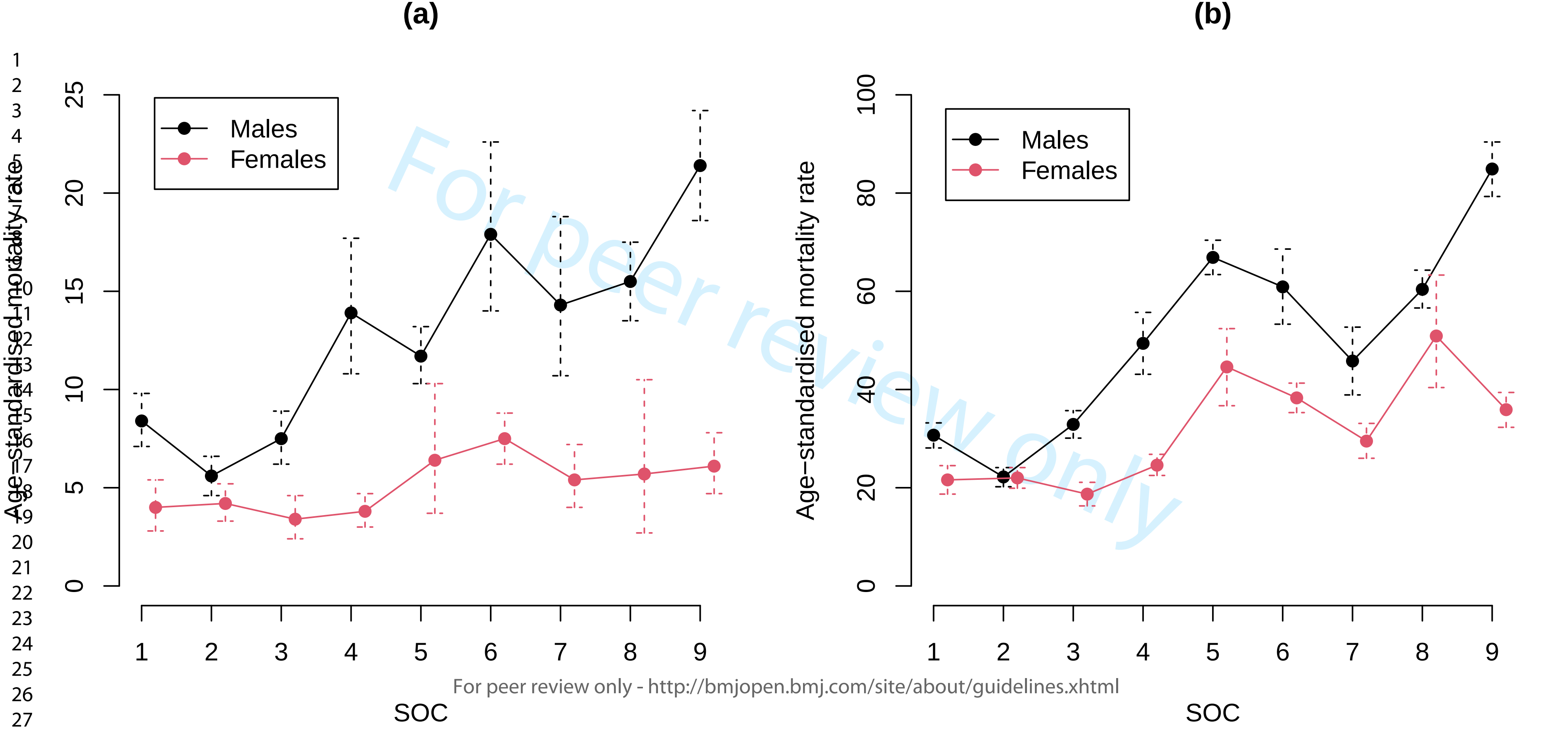

For peer review only

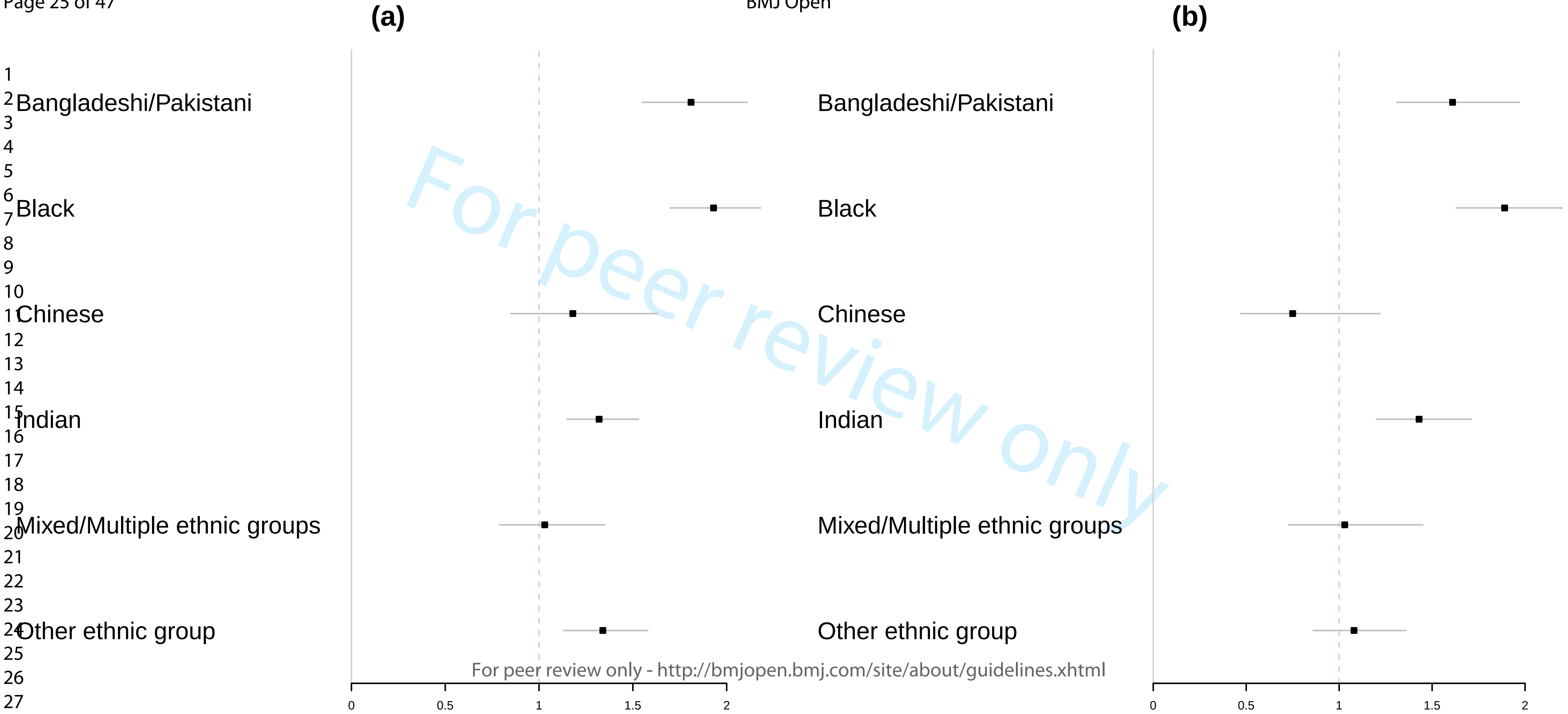

For peer review only

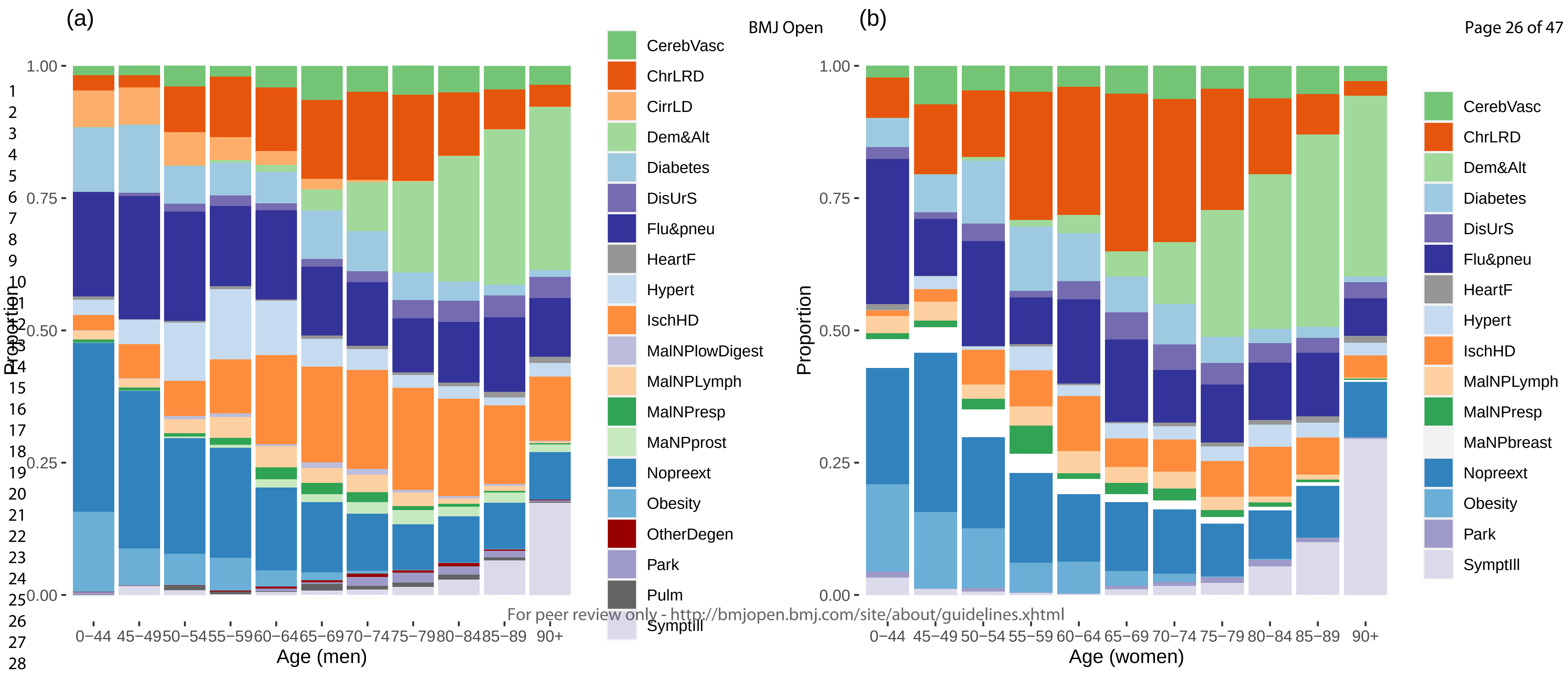
(a)
1.00
0.75
0.50
0.25
0.00
Proportion
0−44
45−49
50−54
55−59
60−64
65−69
70−74
75−79
80−84
85−89
90+
Age (men)
CerebVasc
ChrLRD
CirrLD
Dem&Alt
Diabetes
DisUrS
Flu&pneu
HeartF
Hypert
IschHD
MalNPlowDigest
MalNPLymph
MalNPresp
MaNPprost
Nopreext
Obesity
OtherDegen
Park
Pulm
SymptIll
(b)
1.00
0.75
0.50
0.25
0.00
Proportion
0−44
45−49
50−54
55−59
60−64
65−69
70−74
75−79
80−84
85−89
90+
Age (women)
CerebVasc
ChrLRD
Dem&Alt
Diabetes
DisUrS
Flu&pneu
HeartF
Hypert
IschHD
MalNPLymph
MalNPresp
MaNPbreast
Nopreext
Obesity
Park
SymptIll

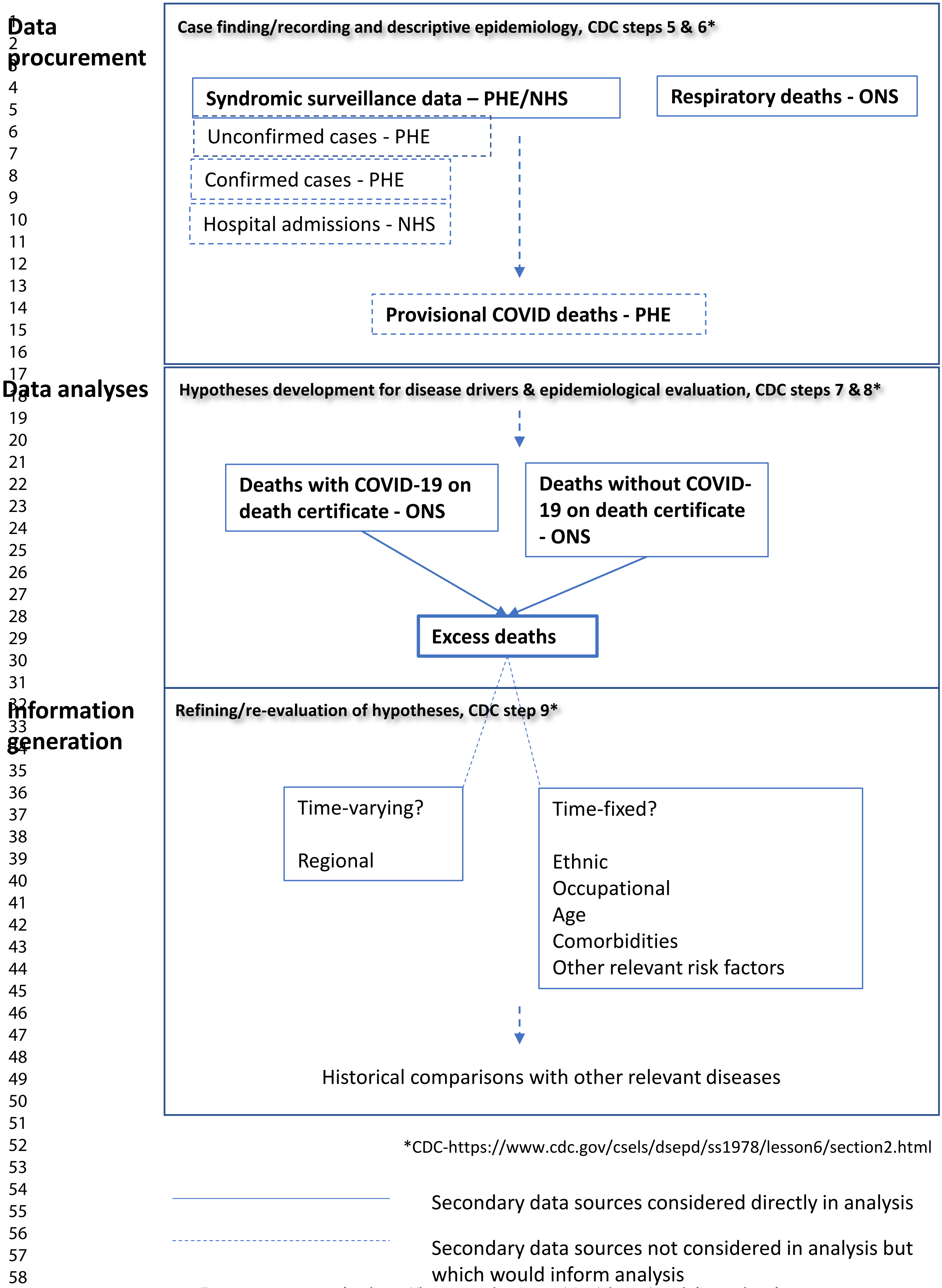
Data procurement
Case finding/recording and descriptive epidemiology, CDC steps 5 & 6*
Syndromic surveillance data – PHE/NHS
Respiratory deaths - ONS
Unconfirmed cases - PHE
Confirmed cases - PHE
Hospital admissions - NHS
Provisional COVID deaths - PHE
Data analyses
Hypotheses development for disease drivers & epidemiological evaluation, CDC steps 7 & 8*
Deaths with COVID-19 on death certificate - ONS
Deaths without COVID-19 on death certificate - ONS
Excess deaths
Information generation
Refining/re-evaluation of hypotheses, CDC step 9*
Time-varying?
Regional
Time-fixed?
Ethnic
Occupational
Age
Comorbidities
Other relevant risk factors
Historical comparisons with other relevant diseases
*CDC-https://www.cdc.gov/csels/dsepd/ss1978/lesson6/section2.html
Secondary data sources considered directly in analysis
Secondary data sources not considered in analysis but which would inform analysis

STROBE Statement—checklist of items that should be included in reports of observational studies (**page numbers relate to clean version of manuscript**)

	Item No.	Recommendation	Page No.	Relevant text from manuscript
**Title and abstract**	1	(*a*) Indicate the study's design with a commonly used term in the title or the abstract	1	Use of routine surveillance data on deaths and illness to provide insight for early-stage UK pandemic planning: lessons from COVID-19
		(*b*) Provide in the abstract an informative and balanced summary of what was done and what was found	1-3	Objectives<br>Reporting of COVID-19 cases, deaths, and testing has often lacked context for appropriate assessment of disease burden within risk groups. The research considers how routine surveillance data might provide initial insights and identify risk factors, setting COVID-19 deaths early in the pandemic into context. This will facilitate the understanding of wider consequences of a pandemic from the earliest stage, reducing fear, aiding in accurately assessing disease burden and in ensuring appropriate disease mitigation.<br><br>Setting<br>United Kingdom, 2020.<br><br>Participants<br>The study is a secondary analysis of routine, public-domain, surveillance data and information from Office for National Statistics (ONS), NHS111 and Public Health England (PHE) on deaths and disease.<br><br>Primary and secondary outcome measures<br>Our principal focus is ONS data on deaths mentioning COVID-19 on the death certificate. We also consider information provided in NHS111 and PHE data summaries.<br><br>Results<br>Deaths with COVID-19 significantly contributed to, yet do not entirely explain, abnormally elevated all-cause mortality in the UK from weeks 12-18 of 2020. Early in the UK epidemic COVID-19 was the greatest threat to those with underlying illness, rarely endangering people aged under 40 years. COVID-19 related death rates differed by region, possibly reflecting

For peer review only

				underlying population structure. Risk of COVID-19-related death was greater for health and social care staff and BAME individuals, having allowed for documented risk factors.<br><br>Conclusion<br>Early contextualisation of public health data is critical to recognising who gets sick, when, and why. Understanding at-risk groups facilitates a targeted response considering indirect consequences of society's reaction to a pandemic alongside disease-related impacts. COVID-19related deaths mainly mirror historical patterns, and excess non-COVID-19 related deaths partly reflect reduced access to and uptake of healthcare during lockdown. Future outbreak response will improve through better understanding of connectivity between disease monitoring systems to aid interpretation of disease risk patterns, facilitating nuanced mitigation measures.
**Introduction**				
Background/rationale	2	Explain the scientific background and rationale for the investigation being reported	4	Intense media reporting during the COVID-19 pandemic has focused on presenting daily data on cases, deaths and testing associated with the virus. The pandemic has undoubtedly changed our world - governments have employed unprecedented (in our times) lockdown methods to reduce transmission. These measures have greatly impacted society. The regular reporting of daily COVID-19 infections and deaths has alarmed the public, particularly when understanding of risk factors dictating severity of COVID-19 symptoms is only slowly emerging. The global population is immunologically naïve to this emerging pathogen, and society, at the time, had no available specific mitigation measures including immunological therapies, other than hand washing, social distancing, mask wearing etc. Clinical and support staff in hospitals, health and social care staff in care homes and other settings, and key workers in transport and infrastructure industries were at increased risk of contracting the disease[1] due to frequent contact with people with high viral loads and the high aerosolized and fomite transmission potential of this virus[2]. Early analyses in England and Wales identified main risk factors for death from COVID-19 including older age, deprivation, and comorbidities[3], but did not consider how this risk differed from 'typical' all-cause mortality among these groups. This baseline comparison is vital to understanding what additional risk is posed by COVID-19, and to whom. Excess mortality from COVID-19 in the UK has been modelled controlling for underlying conditions and age[4], and some conditions such as

For peer review only - http://bmjopen.bmj.com/site/about/guidelines.xhtml

For peer review only

				uncontrolled diabetes and severe asthma are associated with death[3]. However, understanding of the health loss impacts of COVID-19 is still limited by a lack of contextualising information, reducing our ability to respond to the challenges this disease poses, both directly and indirectly, in a proportionate, targeted manner.<br><br>We provide context for deaths and disease from COVID-19, by comparing these against a historical benchmark of when, who, and how people become ill and died pre-COVID-19. Examining associations between poor COVID-19 outcomes, demographic and socio-economic differences, age, sex and comorbidities in the context of 'usual' population health structures, enhances understanding of specific risk groups and hence has a role to play in maximising the effectiveness of risk mitigation strategies whilst minimising the likelihood of unnecessary and undesirable impacts. Examining excess deaths (above that normally expected at a time-point) is, furthermore, important for interpreting the total impact of a pandemic. Syndromic surveillance describing clinical symptoms and healthcare use is scrutinised to supplement clinical surveillance information used to populate the COVID-19 epidemic curve.
Objectives	3	State specific objectives, including any prespecified hypotheses	1-2	Reporting of COVID-19 cases, deaths, and testing has often lacked context for appropriate assessment of disease burden within risk groups. The research considers how routine surveillance data might be used to provide initial insights in a pandemic and identify risk factors, setting COVID-19 deaths early on in the pandemic into context. This will facilitate the anticipation of wider consequences of a pandemic from the earliest stage, reducing fear, aiding in accurately assessing disease burden and in ensuring appropriate disease mitigation.
**Methods**				
Study design	4	Present key elements of study design early in the paper	5	**Study is concerned with addressing the importance of context to numbers and study is designed accordingly.**<br>***In text:***<br>All data must be viewed in their proper context before patterns can be inferred, and in this setting, against a historical baseline. In each case, profiles for COVID-19 deaths were considered against systematic differences in historical disease rates from appropriate comparison populations, to identify when disease was in excess of expected rates.

For peer review only

For peer review only - http://bmjopen.bmj.com/site/about/guidelines.xhtml

Setting	5	Describe the setting, locations, and relevant dates, including periods of recruitment, exposure, follow-up, and data collection	1	United Kingdom, 2020.
Participants	6	(*a*) *Cross-sectional study*—Give the eligibility criteria, and the sources and methods of selection of participants	5	**All deaths involving COVID and comparison with baseline categories under study**: Causes of death were defined using the International Classification of Diseases, 10th Revision (ICD-10)[5]. Deaths involving COVID-19 were defined as those with an underlying cause, or any mention, of ICD-10 codes U07.1 (COVID-19, virus identified) or U07.2 (COVID-19, virus not identified) on the death certificate. All causes of death is the total number of deaths registered during the same time period including those involving COVID-19.<br><br>The baseline comparison group to examine weekly temporal variation in COVID-19 deaths was deaths from respiratory disease across a historical ten-year period (2010 to 2019 inclusive).
Variables	7	Clearly define all outcomes, exposures, predictors, potential confounders, and effect modifiers. Give diagnostic criteria, if applicable		**The outcome consistently throughout is death with COVID-19 against a comparison baseline of context-specific relevance. All outcomes of interest are specified in the methods section. Diagnostic criteria uses a standard ICD-10 classification for deaths from relevant causes.**<br>**In text:**
			5	Causes of death were defined using the International Classification of Diseases, 10th Revision (ICD-10)[5]. Deaths involving COVID-19 were defined as those with an underlying cause, or any mention, of ICD-10 codes U07.1 (COVID-19, virus identified) or U07.2 (COVID-19, virus not identified) on the death certificate. All causes of death is the total number of deaths registered during the same time period including those involving COVID-19.
			4-5	When considering data regionally, rates of death per million population was the primary focus: this allowed for different population sizes within regions and hence created a metric which is comparable across geographies. Here, the mean number of deaths per million population across the previous 5-year period was used as the baseline comparison. Deaths associated with COVID-19 and excess deaths (deaths which do not attribute COVID-19 on the death certificate) were both reported.

For peer review only - http://bmjopen.bmj.com/site/about/guidelines.xhtml

				Rates were again used to compare the risks associated with different Standard Occupational Categories (SOCs) for individuals between 20 and 64 years of age. Age-standardised rates per 100,000 population, standardised to the 2013 European Standard Population, were used in each category to correct for different numbers of people from different age groups working in each group, to ensure comparability between groups. Again the focus is upon the early part of the pandemic, with deaths registered up to and including 20th April 2020 constituting the data. Comparison with deaths from all causes occurring in these categories within the same timeframe creates a natural baseline for deciding how the rate of people dying with COVID-19 in a certain SOC compares with the rate of death in general in that SOC, and helps to distinguish specific COVID-related effects from more subtle societal impacts which might be influencing death rates more generally. Empirical 95% confidence intervals were provided to facilitate comparisons.<br><br>To examine the effects in black and minority ethnic (BAME) groups, again early pandemic data from 2nd March to 10th April 2020 inclusive were considered. Odds ratios are used to compare categories; these were calculated by the ONS using logistic regression models which correct for age (in 5 year categories); rural or urban inhabitants; IMD decile; socio-economic status; and self-reported health and activity status. Forest plots were used to show the estimated odds ratios for dying in each category; 95% confidence intervals were also represented.<br><br>Finally, the representation of co-morbidities amongst COVID-19 deaths in March and April 2020 was explored graphically using a stacked bar chart of the proportion of males and females separately reporting each co-morbidity across age categories (including 0-44 years; 45 to 49 years; 50 to 54 years; 55 to 59 years; 60 to 64 years; 65 to 69 years; 70 to 74 years; 75 to 79 years; 80 to 84 years; 85 to 89 years; and 90 years plus). This allows immediate comparison of how the profile of co-morbidities changes in general by age; whether different comorbidities are more readily apparent in males and females; and whether the evolution of comorbidities as age increases, differs for the two sexes.
Data sources/ measurement	8*	For each variable of interest, give sources of data and details of methods of assessment (measurement). Describe comparability of	*5*	**Data sources**<br>The principal data source was the Office for National Statistics (ONS); dashboards from Public Health England (PHE) and syndromic surveillance in England via NHS 111 were

For peer review only

		assessment methods if there is more than one group		additionally consulted. Primary focus for the analyses presented was the ONS data, which provide gold-standard confirmed recorded causes of death for UK residents. Use of ONS data is licensed under the Open Government Licence v.3.0.
Bias	9	Describe any efforts to address potential sources of bias	15	**Surveillance data biases thoroughly described:** Any analysis based upon surveillance data is subject to limitations. Biases in surveillance data are well-known and well-documented[26]. Data on cases of disease is informative, but can be heavily biased by who appears in the system, and why. For example, any estimate of the case fatality ratio for COVID-19 from the early part of the pandemic would potentially be over-estimated as a consequence of the likely huge under-ascertainment of disease in the early stages, when knowledge about COVID-19 was evolving, and testing was largely limited to hospital cases of disease (the most severe manifestations). It is for this reason that theis research in this paper has focused on data from the ONS, which records conclusive cause of death and is the most complete and accurate resource for UK deaths which should ensure that any biases of reporting are as minimminimised.al as they can be. information of surveillance data.
Study size	10	Explain how the study size was arrived at	5	**Study covers all death data as reported by the ONS in the specified time window so this is a census, sample size calculation not needed:** ***In text:*** Data sources The principal data source was the Office for National Statistics (ONS); dashboards from Public Health England (PHE) and syndromic surveillance in England via NHS 111 were additionally consulted. Primary focus for the analyses presented was the ONS data, which provide gold-standard confirmed recorded causes of death for UK residents. Use of ONS data is licensed under the Open Government Licence v.3.0.

Continued on next page

For peer review only - http://bmjopen.bmj.com/site/about/guidelines.xhtml

Quantitative variables	11	Explain how quantitative variables were handled in the analyses. If applicable, describe which groupings were chosen and why	5-6	**Various descriptive quantitative methods used and described in the methods section:** The baseline comparison group to examine weekly temporal variation in COVID-19 deaths was deaths from respiratory disease across a historical ten-year period (2010 to 2019 inclusive). The mean number of respiratory deaths in weeks 1 to 15 of the year, together with an empirical 95% confidence interval, was calculated and plotted against the numbers of COVID deaths across this same time period in 2020.<br><br>When considering data regionally, rates of death per million population was the primary focus: this allowed for different population sizes within regions and hence created a metric which is comparable across geographies. Here, the mean number of deaths per million population across the previous 5-year period was used as the baseline comparison. Deaths associated with COVID-19 and excess deaths (deaths which do not attribute COVID-19 on the death certificate) were both reported.<br><br>Rates were again used to compare the risks associated with different Standard Occupational Categories (SOCs) for individuals between 20 and 64 years of age. Age-standardised rates per 100,000 population, standardised to the 2013 European Standard Population, were used in each category to correct for different numbers of people from different age groups working in each group, to ensure comparability between groups. Again the focus is upon the early part of the pandemic, with deaths registered up to and including 20th April 2020 constituting the data. Comparison with deaths from all causes occurring in these categories within the same timeframe creates a natural baseline for deciding how the rate of people dying with COVID-19 in a certain SOC compares with the rate of death in general in that SOC, and helps to distinguish specific COVID-related effects from more subtle societal impacts which might be influencing death rates more generally. Empirical 95% confidence intervals were provided to facilitate comparisons.<br><br>To examine the effects in black and minority ethnic (BAME) groups, again early pandemic data from 2nd March to 10th April 2020 inclusive were considered. Odds ratios are used to compare categories; these were calculated by the ONS using logistic regression models which correct for age (in 5 year categories); rural or urban inhabitants; IMD decile; socio-economic status; and self-reported health and activity status. Forest plots were used to show the

For peer review only - http://bmjopen.bmj.com/site/about/guidelines.xhtml

				estimated odds ratios for dying in each category; 95% confidence intervals were also represented.<br><br>Finally, the representation of co-morbidities amongst COVID-19 deaths in March and April 2020 was explored graphically using a stacked bar chart of the proportion of males and females separately reporting each co-morbidity across age categories (including 0-44 years; 45 to 49 years; 50 to 54 years; 55 to 59 years; 60 to 64 years; 65 to 69 years; 70 to 74 years; 75 to 79 years; 80 to 84 years; 85 to 89 years; and 90 years plus). This allows immediate comparison of how the profile of co-morbidities changes in general by age; whether different comorbidities are more readily apparent in males and females; and whether the evolution of comorbidities as age increases, differs for the two sexes.
Statistical methods	12	(*a*) Describe all statistical methods, including those used to control for confounding	6	**Confounding controlled via number of approaches: stratification widely used and many outcomes are controlled for confounders and extra explanatory variables at source.**<br>In text:<br>Rates were again used to compare the risks associated with different Standard Occupational Categories (SOCs) for individuals between 20 and 64 years of age. Age-standardised rates per 100,000 population, standardised to the 2013 European Standard Population, were used in each category to correct for different numbers of people from different age groups working in each group, to ensure comparability between groups. Again the focus is upon the early part of the pandemic, with deaths registered up to and including 20th April 2020 constituting the data. Comparison with deaths from all causes occurring in these categories within the same timeframe creates a natural baseline for deciding how the rate of people dying with COVID-19 in a certain SOC compares with the rate of death in general in that SOC, and helps to distinguish specific COVID-related effects from more subtle societal impacts which might be influencing death rates more generally. Empirical 95% confidence intervals were provided to facilitate comparisons.<br><br>To examine the effects in black and minority ethnic (BAME) groups, again early pandemic data from 2nd March to 10th April 2020 inclusive were considered. Odds ratios are used to compare categories; these were calculated by the ONS using logistic regression models which correct for age (in 5 year categories); rural or urban inhabitants; IMD decile; socio-economic status; and self-reported health and activity status. Forest plots were used to show the

For peer review only

		estimated odds ratios for dying in each category; 95% confidence intervals were also represented.
(*b*) Describe any methods used to examine subgroups and interactions	5-6	**Various subgroups are considered in the analysis (comorbidities, ethic groupings, SOCs) and a combination of graphical and numerical descriptive analysis was used.**<br>**In text:**<br>When considering data regionally, rates of death per million population was the primary focus: this allowed for different population sizes within regions and hence created a metric which is comparable across geographies. Here, the mean number of deaths per million population across the previous 5-year period was used as the baseline comparison. Deaths associated with COVID-19 and excess deaths (deaths which do not attribute COVID-19 on the death certificate) were both reported.<br><br>Rates were again used to compare the risks associated with different Standard Occupational Categories (SOCs) for individuals between 20 and 64 years of age. Age-standardised rates per 100,000 population, standardised to the 2013 European Standard Population, were used in each category to correct for different numbers of people from different age groups working in each group, to ensure comparability between groups. Again the focus is upon the early part of the pandemic, with deaths registered up to and including 20th April 2020 constituting the data. Comparison with deaths from all causes occurring in these categories within the same timeframe creates a natural baseline for deciding how the rate of people dying with COVID-19 in a certain SOC compares with the rate of death in general in that SOC, and helps to distinguish specific COVID-related effects from more subtle societal impacts which might be influencing death rates more generally. Empirical 95% confidence intervals were provided to facilitate comparisons.<br><br>To examine the effects in black and minority ethnic (BAME) groups, again early pandemic data from 2nd March to 10th April 2020 inclusive were considered. Odds ratios are used to compare categories; these were calculated by the ONS using logistic regression models which correct for age (in 5 year categories); rural or urban inhabitants; IMD decile; socio-economic status; and self-reported health and activity status. Forest plots were used to show the estimated odds ratios for dying in each category; 95% confidence intervals were also represented.

For peer review only

				Finally, the representation of co-morbidities amongst COVID-19 deaths in March and April 2020 was explored graphically using a stacked bar chart of the proportion of males and females separately reporting each co-morbidity across age categories (including 0-44 years; 45 to 49 years; 50 to 54 years; 55 to 59 years; 60 to 64 years; 65 to 69 years; 70 to 74 years; 75 to 79 years; 80 to 84 years; 85 to 89 years; and 90 years plus). This allows immediate comparison of how the profile of co-morbidities changes in general by age; whether different comorbidities are more readily apparent in males and females; and whether the evolution of comorbidities as age increases, differs for the two sexes..
		(*c*) Explain how missing data were addressed	NA	**The ONS deaths data is the most complete census of causes of death in the UK and this is the main focus of our analysis. We do not try to re-estimate missing deaths since this is not the focus of this paper.**
		(*d*) If applicable, describe analytical methods taking account of sampling strategy	NA	**The data are a census of deaths so the sampling strategy is to examine all deaths which are confirmed, and hence analytical methods taking account of a sampling strategy are not required.**
		(*e*) Describe any sensitivity analyses	NA	**No cut-offs etc. are used and formal statistical analysis is minimal (approaches largely descriptive). We initially looked at unconfirmed deaths (Figure 6) but these provided less useful numerators so focussed on confirmed deaths.**
**Results**				
Participants	13*	(a) Report numbers of individuals at each stage of study—eg numbers potentially eligible, examined for eligibility, confirmed eligible, included in the study, completing follow-up, and analysed	7-10	**Numbers of individuals are reported for all analyses and eligibility criteria are clearly specified. As this is a census-based study of deaths, by definition a final endpoint, the issue of completing follow-up is not relevant here.**<br>**In text:**<br>Temporal variation in COVID-19 deaths<br>The number of deaths from all causes varies annually and seasonally, peaking in winter. Typically, respiratory deaths range from 10% to 22% of all deaths and are seasonal, peaking annually in January; the 2015 peak was high (16,237 deaths in week two compared to an average of 12,277 deaths that week over the previous five years) due to a severe influenza season, and 2018 similarly had a severe influenza season resulting in a high death count. The minimum number of weekly deaths over this five year period was 6,606 (week 54, 2013). In 2020 deaths from respiratory infections were lower than the mean in the previous five years

For peer review only - http://bmjopen.bmj.com/site/about/guidelines.xhtml

until early April (week 14), after which they became higher than historical rates when including deaths from COVID-19 (Figure 1a) (in week 14 observed respiratory deaths exceeded the 5-year historical upper 95% confidence interval limit by 146). An excess of unexplained deaths becomes clear from week 14 onwards (Figure 1b). Following a period of excess deaths, in week 25 of 2020, for the first time there were fewer deaths than the equivalent previous 5 year average (65 fewer deaths), and similarly in weeks 26 through to 28 there were 917 fewer deaths than the total of the averages across years for those weeks in the previous five years[8].

Regional differences in COVID-19 deaths

Regions of England and Wales experience different death rates and this pattern is true for deaths from COVID-19 (Figure 2) e.g. rates were highest and peaked in week 17 in London (204 per million), the North-West (185 per million), the North-East (179 per million), and the West Midlands (169 per million). Peak rates were lowest in the South-West (95 per million) and East Midlands (116 per million). From weeks 13-18 (March 23-May 3), all regions of England and Wales experienced excess non-COVID-19 related deaths. This was most apparent in the West Midlands in week 17 (starting April 20), with a peak of approximately 91 deaths per million. Between weeks 13-18 (March 23- May 3) there were 46,594 excess deaths in England and Wales, 13,399 of which were listed as non-COVID-19-related. In week 25 (19th June) the total deaths dipped below the 5-year historical average for the first time (9,339 compared to 9,404) and this pattern continued until 10th July.

Occupational differences in COVID-19 deaths

After age-standardisation (rates per 100,000 population, standardised to the 2013 European Standard Population), men employed in lower skilled occupations (21.4, 95% CI 18.6-24.2) (Figure 3a) were more likely to die of COVID-related illness (k=225 deaths from n=1321 deaths in total across occupations for men[9]), as was true for all-cause mortality (Figure 3b, k=915 deaths out of n=5627 deaths). This differs for women, where those employed as carers in health and social care, leisure and other service operations (Figure 3a) were most likely to die from COVID-related illness (k=130 deaths out of n=531 deaths in total across occupations for women), but not more likely to die if examining all-cause mortality (Figure 3b, k=651 deaths from n=3003 deaths). For both men and women, the less technical and more manual their occupation (using ONS SOC 2020 categories), the greater the risk of dying from any

For peer review only - http://bmjopen.bmj.com/site/about/guidelines.xhtml

cause including COVID-19-related disease. In addition, when occupations are more manual, variation in age-standardised mortality rates is higher, particularly for men or women in certain SOC categories e.g. men undertaking administrative and secretarial roles; women in skilled trades; men in caring, leisure and other service occupations; men in sales and customer service roles; women working as process, plant and machine operatives; and men undertaking low skilled elementary roles.

A crude comparison suggests that age-standardised mortality rates for most occupations are reduced by COVID-19 relative to deaths from any cause (Table 1), with the rate only increased (for both sexes) in caring, leisure and other service occupations.

Ethnic associations with COVID-19 deaths

As previously reported by the ONS[10] in data from 2nd March to 10th April 2020, there were increased odds of dying from COVID-19 for Bangladeshi/Pakistani (386 deaths), Black (766 deaths) and Indian (483 deaths) ethnic groups (for both sexes) when compared with a baseline white group and adjusted for age, region, rural/urban, Index of Multiple Deprivation decile, household composition, socio-economic status and underlying health conditions. In total across all groups, in this time period 12,805 deaths occurred. For Chinese and mixed ethnic groups the odds ratio was not statistically significantly different from one, perhaps due to small sample size (59 deaths in total observed in Chinese ethnic groups in this time period, from a total of 12,805 across all categories).

FIGURE 4a AND 4b HERE.

Impact of comorbidities upon COVID-19 deaths

Deaths related to COVID-19 reflect broad underlying patterns, with more reported in men (61.3%, n=6,342) and older people (at week 15, 87% (n=8,985) of deaths were in those aged over 65, 69% (n=7,135) were in people aged over 75). Data from the ONS across 2019 show an increased proportion of health conditions (chest and breathing issues and heart/blood pressure/circulatory problems) related to age. The percentages of people with heart, blood pressure, or circulatory problems were 0.48% (16-19 years); 6.31% (20-39 years); 31.35% (40-59 years); and 61.86% (60+ years). Similarly, the percentages of people with chest and breathing problems were 4.85% (16-19 years); 26.96% (20-39 years); 32.23% (40-59 years);

For peer review only - http://bmjopen.bmj.com/site/about/guidelines.xhtml

				and 35.96% (60+ years)[11]. Long-term comorbidities such as ischaemic heart disease and hypertensive disease are commonly present in men dying with COVID-19 (Figure 5a), particularly in higher age groups; a similar pattern was observed for cerebrovascular diseases in women (Figure 5b). As people reach very advanced age, for both sexes the predominant comorbidities are dementia and Alzheimer's (Figures 5a and 5b).
		(b) Give reasons for non-participation at each stage	NA	**This is a census-based study involving an analysis of public domain data covering the endpoint that is death, and so non-participation is not a consideration.**
		(c) Consider use of a flow diagram	NA	**We do not believe a flow diagram will bring any useful information to this piece, since all eligible individuals are included.**
Descriptive data	14*	(a) Give characteristics of study participants (eg demographic, clinical, social) and information on exposures and potential confounders	7-10	**The aggregate characteristics of people dying with COVID by comparison with appropriate baselines is the main thrust of this paper, hence much detail in the manuscript is devoted to this and is reported in the results and in Figures 2, 3, 4 and 5 and Table 1.**<br>***In text:***<br>Regional differences in COVID-19 deaths<br>Regions of England and Wales experience different death rates and this pattern is true for deaths from COVID-19 (Figure 2) e.g. rates were highest and peaked in week 17 in London (204 per million), the North-West (185 per million), the North-East (179 per million), and the West Midlands (169 per million). Peak rates were lowest in the South-West (95 per million) and East Midlands (116 per million). From weeks 13-18 (March 23-May 3), all regions of England and Wales experienced excess non-COVID-19 related deaths. This was most apparent in the West Midlands in week 17 (starting April 20), with a peak of approximately 91 deaths per million. Between weeks 13-18 (March 23- May 3) there were 46,594 excess deaths in England and Wales, 13,399 of which were listed as non-COVID-19-related. In week 25 (19th June) the total deaths dipped below the 5-year historical average for the first time (9,339 compared to 9,404) and this pattern continued until 10th July.<br><br>Occupational differences in COVID-19 deaths<br>After age-standardisation (rates per 100,000 population, standardised to the 2013 European Standard Population), men employed in lower skilled occupations (21.4, 95% CI 18.6-24.2) (Figure 3a) were more likely to die of COVID-related illness (k=225 deaths from n=1321

For peer review only - http://bmjopen.bmj.com/site/about/guidelines.xhtml

deaths in total across occupations for men[9]), as was true for all-cause mortality (Figure 3b, k=915 deaths out of n=5627 deaths). This differs for women, where those employed as carers in health and social care, leisure and other service operations (Figure 3a) were most likely to die from COVID-related illness (k=130 deaths out of n=531 deaths in total across occupations for women), but not more likely to die if examining all-cause mortality (Figure 3b, k=651 deaths from n=3003 deaths). For both men and women, the less technical and more manual their occupation (using ONS SOC 2020 categories), the greater the risk of dying from any cause including COVID-19-related disease. In addition, when occupations are more manual, variation in age-standardised mortality rates is higher, particularly for men or women in certain SOC categories e.g. men undertaking administrative and secretarial roles; women in skilled trades; men in caring, leisure and other service occupations; men in sales and customer service roles; women working as process, plant and machine operatives; and men undertaking low skilled elementary roles.

A crude comparison suggests that age-standardised mortality rates for most occupations are reduced by COVID-19 relative to deaths from any cause (Table 1), with the rate only increased (for both sexes) in caring, leisure and other service occupations.

Ethnic associations with COVID-19 deaths

As previously reported by the ONS[10] in data from 2nd March to 10th April 2020, there were increased odds of dying from COVID-19 for Bangladeshi/Pakistani (386 deaths), Black (766 deaths) and Indian (483 deaths) ethnic groups (for both sexes) when compared with a baseline white group and adjusted for age, region, rural/urban, Index of Multiple Deprivation decile, household composition, socio-economic status and underlying health conditions. In total across all groups, in this time period 12,805 deaths occurred. For Chinese and mixed ethnic groups the odds ratio was not statistically significantly different from one, perhaps due to small sample size (59 deaths in total observed in Chinese ethnic groups in this time period, from a total of 12,805 across all categories).

Impact of comorbidities upon COVID-19 deaths

Deaths related to COVID-19 reflect broad underlying patterns, with more reported in men (61.3%, n=6,342) and older people (at week 15, 87% (n=8,985) of deaths were in those aged

For peer review only - http://bmjopen.bmj.com/site/about/guidelines.xhtml

				over 65, 69% (n=7,135) were in people aged over 75). Data from the ONS across 2019 show an increased proportion of health conditions (chest and breathing issues and heart/blood pressure/circulatory problems) related to age. The percentages of people with heart, blood pressure, or circulatory problems were 0.48% (16-19 years); 6.31% (20-39 years); 31.35% (40-59 years); and 61.86% (60+ years). Similarly, the percentages of people with chest and breathing problems were 4.85% (16-19 years); 26.96% (20-39 years); 32.23% (40-59 years); and 35.96% (60+ years)[11]. Long-term comorbidities such as ischaemic heart disease and hypertensive disease are commonly present in men dying with COVID-19 (Figure 5a), particularly in higher age groups; a similar pattern was observed for cerebrovascular diseases in women (Figure 5b). As people reach very advanced age, for both sexes the predominant comorbidities are dementia and Alzheimer's (Figures 5a and 5b).
		(b) Indicate number of participants with missing data for each variable of interest	NA	**Again as this is a census-based study involving an analysis of public domain data covering the endpoint that is death this is not relevant to our work.**
Outcome data	15*	*Cross-sectional study*—Report numbers of outcome events or summary measures	NA	**The outcome measure throughout is number of deaths and this is referenced in numerous places and visualised graphically in Figures throughout the manuscript.**
Main results	16	(*a*) Give unadjusted estimates and, if applicable, confounder-adjusted estimates and their precision (eg, 95% confidence interval). Make clear which confounders were adjusted for and why they were included	9	**Various adjusted and unadjusted estimates are presented, for ethnic groupings, SOCs, comorbidities etc. throughout the results sections (pages 7-9). Ethnic analysis, for example, adjusts at source (ONS analysis) for Age, region, rural/urban, IMD decile, household composition, socio-economic status, health.**<br>**In text:**<br>Ethnic associations with COVID-19 deaths<br>As previously reported by the ONS[10] in data from 2nd March to 10th April 2020, there were increased odds of dying from COVID-19 for Bangladeshi/Pakistani (386 deaths), Black (766 deaths) and Indian (483 deaths) ethnic groups (for both sexes) when compared with a baseline white group and adjusted for age, region, rural/urban, Index of Multiple Deprivation decile, household composition, socio-economic status and underlying health conditions. In total across all groups, in this time period 12,805 deaths occurred. For Chinese and mixed ethnic groups the odds ratio was not statistically significantly different from one, perhaps due to small sample size (59 deaths in total observed in Chinese ethnic groups in this time period, from a total of 12,805 across all categories).

For peer review only - http://bmjopen.bmj.com/site/about/guidelines.xhtml

	(*b*) Report category boundaries when continuous variables were categorized	NA	**We have not categorised any continuous variables in the analysis: all data are analysed as reported**
	(*c*) If relevant, consider translating estimates of relative risk into absolute risk for a meaningful time period	NA	**None of our calculations produce relative risks and so this is not relevant here.**

Continued on next page

For peer review only

Other analyses	17	Report other analyses done—eg analyses of subgroups and interactions, and sensitivity analyses	NA	**We have comprehensively covered all analyses and results in the above points.**
**Discussion**				
Key results	18	Summarise key results with reference to study objectives	10-12	This analysis characterises the COVID-19 pandemic in England and Wales in the context of excess death over time, by region, and risk factor. Increases in mortality in April were predominantly driven by COVID-19, but non-COVID-19 excess deaths also increased in April-May 2020 across all regions. Compared to historical rates of death amongst occupational groups, COVID-19 related deaths generally followed normal patterns, excepting individuals among caring, leisure, and other service occupations who were more likely to die from COVID-related illness than die from any illness. Rates of death from COVID-19 related illness are higher among Black and Ethnic Minority (BAME) populations, but small sample sizes preclude all-cause mortality comparisons. Finally, pre-existing comorbidities are a strong risk factor for COVID-19 related death and are more common among men and the elderly, explaining why these groups appear to be at excess risk of death related to COVID-19. Thus, patterns of death and excess death from COVID-19 mirror historical trends in mortality. This contextualisation of COVID-19 is critical to inform plans to protect the vulnerable while helping low risk populations in society to resume more normal lifestyle patterns.<br><br>The lower-than-expected death toll from week 25 onwards may be suggestive of a mortality displacement ('harvesting') impact; a proportion of the population who died at the epidemic peak (weeks 13 to 18) may have died in the shorter-term in other circumstances. The complete picture is likely to be far more complex, but the harvesting phenomenon is previously described e.g. due to impacts of heatwaves and cold spells[15] and influenza in 1918/19 (compared to deaths from tuberculosis)[16]. Such population readjustments need to be taken into account in planning processes as the overall health loss may be relatively small compared to a disease or health problem that kills people who are healthy.<br>Context to age and gender<br>Much age and gender-related health risk is more appropriately attributable to increased prevalence of underlying comorbidities. We are more likely to die as we age, with 84% of annual deaths in people over 65 years, and 66% in those over 75 years[17]. Men also die

For peer review only - http://bmjopen.bmj.com/site/about/guidelines.xhtml

earlier in most age groups and have lower life expectancies (79.2 years) than women (82.9 years)[18]. As we age our likelihood of having long-term illness increases as has been discussed. Though the burden of risk from COVID-19 lies with older age groups, more thorough epidemiological analysis may identify some subpopulations that could be classified as lower (or higher) risk. Such analysis would inform better risk management strategies, allowing mobility and economic activity amongst some low-risk older populations, as well as intrinsically low risk groups such as young people.

Context to comorbidities

Patterns of co-morbidities for COVID-19-related deaths mirror the increase in these diseases with age (in non-COVID circumstances) e.g. ischaemic heart disease is more frequently experienced with age by men than women[19]. It is unclear whether an increasing representation of dementia and Alzheimer's as comorbidities is seen because they are genuine comorbidities in their own right or due to data biases. The most important other comorbidities are chronic lower respiratory disease in females and ischaemic heart disease in males. A role for specific genes linked to dementia and Alzheimer's and poor COVID-19 response has recently been suggested[20] and warrants further investigation. It has not been possible to locate for 2020, the numbers of deaths by each comorbidity in its own right: these would be useful for comparison and establishment of any excess, but whether an excess of deaths with COVID-19 by any of the comorbidities will occur is unclear at the time of writing.

Context to ethnicity and occupation

Ethnicity and occupation are common risk factors for morbidity and mortality from infectious disease, but are not often reported in surveillance data[21]. Heightened reported risks among specific ethnic and occupational groups are alarming, and COVID-19 has brought renewed attention to health disparities inherent in the UK population, but excepting care, service, and leisure workers, the precise nature and drivers of excess COVID-19 risk in different groups remains unclear. When considering occupational risk for example, age-standardised mortality ratios (ASMRs) in different occupational categories for COVID-19 mortality must be considered alongside ASMRs for all-cause mortality. For example, when the COVID-19-associated ASMR in an occupational category is high relative to deaths from all causes, this suggests COVID-19-associated impacts should be considered in managing return to work.

For peer review only - http://bmjopen.bmj.com/site/about/guidelines.xhtml

				Consequences of COVID-19 and our response to its presence<br>The reduction in accident and emergency consultations is inconsistent with the pattern observed in 2019; it suggests a reluctance or inability of the public to access healthcare during lockdown. Unfortunately, comparisons against a longer range of historical data are not possible since the surveillance system changed in 2018, with greater numbers of hospitals reporting to the system from this point onwards. The reasons for this reduction may be multi-factorial reflecting reluctance, fear of the virus, and logistical difficulties for GPs. This pattern of reduced healthcare uptake foreshadows an increased health burden as a result of the combination of delays introduced into the system by aspects of both the health services and individuals' responses to COVID-19. However, in the immediate future, a dip in mortality is occurring, compared to baseline. In Wales, where the median age is higher than in any other UK nation or region of England[22], the rate of death per million returned to at, or below, historical levels before any other region in England. This suggests that for high-risk populations (e.g. the elderly), deaths have been compressed within the time window of the pandemic. This phenomenon was previously observed among tuberculosis patients in the months and years following the 1918 Spanish Flu[16]. Thus, continued contextualisation of deaths is critical to accurately assess the long-term impact of COVID-19 on health in the UK - volatility of demand should be considered in resource planning.
Limitations	19	Discuss limitations of the study, taking into account sources of potential bias or imprecision. Discuss both direction and magnitude of any potential bias	14	Methodological limitations<br>Any analysis based upon surveillance data is subject to limitations. Biases in surveillance data are well-known and well-documented[29]. Data on cases of disease is informative but can be heavily biased by who appears in the system, and why. For example, any estimate of the case fatality ratio for COVID-19 from the early part of the pandemic would potentially be over-estimated as a consequence of the likely huge under-ascertainment of disease in the early stages, when knowledge about COVID-19 was evolving, and testing was largely limited to hospital cases of disease (the most severe manifestations). It is for this reason that the research in this paper has focused on data from the ONS, which records conclusive cause of death and is the most complete and accurate resource for UK deaths which should ensure that any biases of reporting are minimised.<br><br>The analysis presented here is largely descriptive, and as such it is not possible to make any statements about, for example, statistical significance of observations. This approach is

For peer review only

				deliberate: it is the authors' intention to demonstrate how a well-chosen graphical display can provide valuable insight, which can be readily interpreted by those without specialist knowledge.
Interpretation	20	Give a cautious overall interpretation of results considering objectives, limitations, multiplicity of analyses, results from similar studies, and other relevant evidence	15	Policy makers have relied on models in the early phase of COVID-19. These must be supported by data-driven evidence on when, where, who and why people get sick and die. Timely emergence and analysis of this information should be used to calibrate social, cultural and economic assessments of the impact of COVID-19 versus our actions to control it, if we are to return to a cautious normality.<br><br>To our knowledge this is the first study to consider reported numbers of COVID-19 illnesses and deaths in England and Wales against their historical disease context. Our research identifies and combines important, open-access data to inform a more nuanced response to emerging disease. Many openly available resources could improve response planning for emerging disease situations such as COVID-19, and could be used to anticipate wider consequences than immediate infection-related impacts. Syndromic surveillance data combined with real-time surveillance would supplement and strengthen the mathematical models informing emerging disease responses. Our analysis highlights the importance of calibrating social, cultural and economic assessments of the direct impact of COVID-19 against potential control actions.
Generalisability	21	Discuss the generalisability (external validity) of the study results	18	In Europe, two surveillance strands are followed and both are restricted access: EU/EEA Member States and the UK report for every 24 hour period the number of laboratory-confirmed cases of COVID-19 using their Early Warning and Response System (EWRS). Enhanced surveillance has also been put in place via The European Surveillance System – TESSy[26]. The restricted access nature of these resources limits their real-time applicability for parties other than those with permitted access. A full consideration of the international picture is beyond the scope of this paper, but the process described herein could be repeated for other populations.
**Other information**				
Funding	22	Give the source of funding and the role of the funders for the present study and, if applicable, for the original study on which the present article is based	16	Funding statement<br>The work was funded through the employment of the authors at the University of Liverpool, UK.

For peer review only

For peer review only - http://bmjopen.bmj.com/site/about/guidelines.xhtml

*Give information separately for cases and controls in case-control studies and, if applicable, for exposed and unexposed groups in cohort and cross-sectional studies.

Note: An Explanation and Elaboration article discusses each checklist item and gives methodological background and published examples of transparent reporting. The STROBE checklist is best used in conjunction with this article (freely available on the Web sites of PLoS Medicine at http://www.plosmedicine.org/, Annals of Internal Medicine at http://www.annals.org/, and Epidemiology at http://www.epidem.com/). Information on the STROBE Initiative is available at www.strobe-statement.org.

For peer review only
